# Resistance of African tropical forests to an extreme climate anomaly

**DOI:** 10.1073/pnas.2003169118

**Published:** 2021-05-17

**Authors:** Amy C. Bennett, Greta C. Dargie, Aida Cuni-Sanchez, John Tshibamba Mukendi, Wannes Hubau, Jacques M. Mukinzi, Oliver L. Phillips, Yadvinder Malhi, Martin J. P. Sullivan, Declan L. M. Cooper, Stephen Adu-Bredu, Kofi Affum-Baffoe, Christian A. Amani, Lindsay F. Banin, Hans Beeckman, Serge K. Begne, Yannick E. Bocko, Pascal Boeckx, Jan Bogaert, Terry Brncic, Eric Chezeaux, Connie J. Clark, Armandu K. Daniels, Thales de Haulleville, Marie-Noël Djuikouo Kamdem, Jean-Louis Doucet, Fidèle Evouna Ondo, Corneille E. N. Ewango, Ted R. Feldpausch, Ernest G. Foli, Christelle Gonmadje, Jefferson S. Hall, Olivier J. Hardy, David J. Harris, Suspense A. Ifo, Kathryn J. Jeffery, Elizabeth Kearsley, Miguel Leal, Aurora Levesley, Jean-Remy Makana, Faustin Mbayu Lukasu, Vincent P. Medjibe, Vianet Mihindu, Sam Moore, Natacha Nssi Begone, Georgia C. Pickavance, John R. Poulsen, Jan Reitsma, Bonaventure Sonké, Terry C. H. Sunderland, Hermann Taedoumg, Joey Talbot, Darlington S. Tuagben, Peter M. Umunay, Hans Verbeeck, Jason Vleminckx, Lee J. T. White, Hannsjoerg Woell, John T. Woods, Lise Zemagho, Simon L. Lewis

**Affiliations:** ^a^School of Geography, University of Leeds, Leeds, LS2 9JT, United Kingdom;; ^b^Department of Environment and Geography, University of York, York, YO10 5NG, United Kingdom;; ^c^Department of Geography, University College London, London, WC1E 6BT, United Kingdom;; ^d^Service of Wood Biology, Royal Museum for Central Africa, Tervuren, 3080 Belgium;; ^e^Faculté de Gestion de Ressources Naturelles Renouvelables, Université de Kisangani, Kisangani, R408, Democratic Republic of Congo;; ^f^Faculté des Sciences Appliquées, Université de Mbujimayi, Mbujimayi, Democratic Republic of Congo;; ^g^Department of Environment, Laboratory of Wood Technology, Ghent University, 9000 Ghent, Belgium;; ^h^Democratic Republic of Congo Programme, Wildlife Conservation Society, Kinshasa, Democratic Republic of Congo;; ^i^Salonga National Park, Kinshasa, Democratic Republic of Congo;; ^j^World Wide Fund for Nature, 1196 Gland, Switzerland;; ^k^Environmental Change Institute, School of Geography and the Environment, Oxford University, Oxford, OX1 3QY, United Kingdom;; ^l^Department of Natural Sciences, Manchester Metropolitan University, Manchester, M15 6BH, United Kingdom;; ^m^Forestry Research Institute of Ghana (FORIG), Kumasi, Ghana;; ^n^Mensuration Unit, Forestry Commission of Ghana, Kumasi, Ghana;; ^o^Université Officielle de Bukavu, Bukavu, Democratic Republic of Congo;; ^p^Center for International Forestry Research (CIFOR), Bogor 16115, Indonesia;; ^q^Centre for Ecology and Hydrology, Penicuik, EH26 0QB, United Kingdom;; ^r^Plant Systematic and Ecology Laboratory, Higher Teachers’ Training College, University of Yaounde I, Yaounde, Cameroon;; ^s^Faculté des Sciences et Techniques, Laboratoire de Botanique et Ecologie, Université Marien Ngouabi, Brazzaville, Republic of Congo;; ^t^Isotope Bioscience Laboratory (ISOFYS), Ghent University, 9000 Ghent, Belgium;; ^u^Biodiversity and Landscape Unit, Gembloux Agro-Bio Tech, Université de Liège, 5030 Gembloux, Belgium;; ^v^Congo Programme, Wildlife Conservation Society, Brazzaville, Republic of Congo;; ^w^Rougier-Gabon, Libreville, Gabon;; ^x^Nicholas School of the Environment, Duke University, Durham, NC 27710;; ^y^Forestry Development Authority of the Government of Liberia (FDA), Monrovia, Liberia;; ^z^Faculty of Science, Department of Botany and Plant Physiology, University of Buea, Buea, Cameroon;; ^aa^TERRA Teaching and Research Centre, Forest Is Life, Gembloux Agro-Bio Tech, University of Liège, 5030 Gembloux, Belgium;; ^bb^Agence Nationale des Parcs Nationaux, Libreville, Gabon;; ^cc^Centre de Formation et de Recherche en Conservation Forestiere (CEFRECOF), Epulu, Democratic Republic of Congo;; ^dd^Geography, College of Life and Environmental Sciences, University of Exeter, Exeter, EX4 4QE, United Kingdom;; ^ee^National Herbarium, Yaounde, Cameroon;; ^ff^Forest Global Earth Observatory, Smithsonian Tropical Research Institute, Washington, DC 20560;; ^gg^Evolutionary Biology and Ecology, Faculté des Sciences, Université Libre de Bruxelles, 1050 Bruxelles, Belgium;; ^hh^Royal Botanic Garden Edinburgh, Edinburgh, EH3 5NZ, United Kingdom;; ^ii^École Normale Supérieure, Département des Sciences et Vie de la Terre, Laboratoire de Géomatique et d’Ecologie Tropicale Appliquée, Université Marien Ngouabi, Brazzaville, Republic of Congo;; ^jj^Biological and Environmental Sciences, University of Stirling, Stirling, FK9 4LA, United Kingdom;; ^kk^Department of Environment, Computational & Applied Vegetation Ecology (Cavelab), Ghent University, 9000 Ghent, Belgium;; ^ll^Uganda Programme, Wildlife Conservation Society, Kampala, Uganda;; ^mm^Faculté des Sciences, Laboratoire d’écologie et aménagement forestier, Université de Kisangani, Kisangani, Democratic Republic of Congo;; ^nn^Center for Tropical Conservation, Duke University, Durham, NC 27705;; ^oo^Commission of Central African Forests (COMIFAC), Yaounde, Cameroon;; ^pp^Agence Nationale des Parcs Nationaux, Libreville, Gabon;; ^qq^Ministry of Forests, Seas, Environment and Climate, Libreville, Gabon;; ^rr^Bureau Waardenburg, 4101 CK Culemborg, The Netherlands;; ^ss^Faculty of Forestry, University of British Columbia, Vancouver, BC V6T 1Z4, Canada;; ^tt^Biodiversity International, Yaounde, Cameroon;; ^uu^Institute for Transport Studies, University of Leeds, Leeds, LS2 9JT, United Kingdom;; ^vv^Yale School of Forestry & Environmental Studies, Yale University, New Haven, CT 06511;; ^ww^Wildlife Conservation Society, New York, NY 11224;; ^xx^International Center for Tropical Botany, Department of Biological Sciences, Florida International University, University Park, FL 33199;; ^yy^Faculté des Sciences, Service d’Évolution Biologique et écologie, Université Libre de Bruxelles, 1050 Bruxelles, Belgium;; ^zz^Institut de Recherche en Ecologie Tropicale, Libreville, Gabon;; ^aaa^Sommersbergseestrasse, 8990 Bad Aussee, Austria;; ^bbb^William R. Tolbert, Jr. College of Agriculture and Forestry, University of Liberia, Monrovia, Liberia

**Keywords:** temperature, drought, ENSO, carbon cycle, El Niño

## Abstract

The responses of tropical forests to environmental change are critical uncertainties in predicting the future impacts of climate change. The positive phase of the 2015–2016 El Niño Southern Oscillation resulted in unprecedented heat and low precipitation in the tropics with substantial impacts on the global carbon cycle. The role of African tropical forests is uncertain as their responses to short-term drought and temperature anomalies have yet to be determined using on-the-ground measurements. African tropical forests may be particularly sensitive because they exist in relatively dry conditions compared with Amazonian or Asian forests, or they may be more resistant because of an abundance of drought-adapted species. Here, we report responses of structurally intact old-growth lowland tropical forests inventoried within the African Tropical Rainforest Observatory Network (AfriTRON). We use 100 long-term inventory plots from six countries each measured at least twice prior to and once following the 2015–2016 El Niño event. These plots experienced the highest temperatures and driest conditions on record. The record temperature did not significantly reduce carbon gains from tree growth or significantly increase carbon losses from tree mortality, but the record drought did significantly decrease net carbon uptake. Overall, the long-term biomass increase of these forests was reduced due to the El Niño event, but these plots remained a live biomass carbon sink (0.51 ± 0.40 Mg C ha^−1^ y^−1^) despite extreme environmental conditions. Our analyses, while limited to African tropical forests, suggest they may be more resistant to climatic extremes than Amazonian and Asian forests.

## Significance

The responses of tropical forests to heat and drought are critical uncertainties in predicting the future impacts of climate change. The 2015–2016 El Niño Southern Oscillation (ENSO) resulted in unprecedented heat and low precipitation across the tropics, including in the very poorly studied African tropical forest region. We assess African forest ENSO responses using on-the-ground measurements. Across 100 long-term plots, record high temperatures did not significantly reduce carbon gains from tree growth or significantly increase carbon losses from tree mortality. Overall, despite the climate anomaly, forests continued to gain live biomass over the ENSO period. Our analyses, while limited to African tropical forests, suggest that they may be more resistant to climate extremes than Amazonian and Asian forests.

Tropical forests are a critical component of the global carbon cycle because they are extensive (1), carbon dense (2), and highly productive (3). Therefore, consistent impacts on these forests can have global consequences. Their global importance is seen via atmospheric measurements of CO_2_, showing a near-neutral exchange of carbon across the terrestrial tropics; hence, the large carbon losses from deforestation and degradation are offset by the significant carbon uptake from intact tropical forests and tropical forest regrowth (4). Independently, ground observations of structurally intact old-growth tropical forests also show this uptake, with forest biomass carbon increasing across remaining African (5, 6), Amazonian (7), and Asian (8) forests. Yet, unlike in Amazonia (9, 10) and Asia (8), the impact of a severe drought or a drought and high-temperature event in African tropical forests has never been documented using ground data.

High temperatures test the physiological tolerance of tropical trees. Above optimal temperatures, plants reduce their carbon uptake (11). This includes closing stomata to avoid water loss, reducing internal CO_2_ concentrations, and reducing carbon assimilation in the leaf. Higher temperatures increase vapor pressure deficits (12) and alongside reduced precipitation, increase the chance of hydraulic failure (13). Individually or in combination, these impacts can slow growth and may eventually kill trees (14), although tropical seedling growth can increase with experimental warming (15). As well as reduced carbon uptake, plants use more carbon under higher temperatures; respiration rates tend to increase with short-term increases in temperature both at the leaf (16) and forest stand (17) scales, again reducing tree growth and potentially leading to tree death via carbon starvation (18). Recent analyses of tropical forest plot data showed increased temperatures over the prior 5 y were associated with lower levels of carbon uptake from tree growth and higher levels of carbon loss from tree mortality (6). Furthermore, biome-wide spatial analyses suggest the existence of a temperature threshold above which carbon uptake from tree growth declines rapidly (19). Thus, with high temperature anomalies, we expect reduced tree growth and increased tree mortality.

Drought also impacts trees as water deficits can slow tree growth and if of sufficient strength or duration, can kill trees, either via hydraulic failure or carbon starvation. Hydraulic failure of the xylem has been found across species and biomes in response to drought, while carbon starvation has been documented in some locations including one tropical site (20). Inventory plot observations before, during, and after droughts show the impacts of drought in Asia and Amazonia. In Asia, the 1997 to 1998 El Niño temporarily halted the carbon sink in live biomass in Bornean forests by increasing tree mortality (8, 21). In Amazonia, severe droughts in 2005 and 2010 elevated biomass mortality and in 2010, also significantly reduced tree growth (9, 10). The Amazon biomass carbon sink was reversed by the 2005 drought, and while it rapidly recovered, it is weaker since 2005 (7), potentially due to high-temperature impacts (6). However, while the impacts of short-term drought in their long-term context have been investigated in Amazonia and Asia, in Africa we so far lack any ground-based assessment of large-scale drought impacts due to a paucity of observations.

Although the broad responses of African tropical forests to temperature and drought anomalies might be hypothesized from first principles and the responses of other continents, there are considerable uncertainties. On the one hand, there are grounds for expecting African forests to be especially vulnerable. African forests are already remarkably dry compared with Amazonian and Asian tropical forests, with almost 90% receiving <2,000 mm y^−1^ precipitation (22), the approximate amount necessary to maintain photosynthesis at high levels throughout the year (23). This low rainfall suggests African tropical forests may already be close to their physiological and ecological limits. Additionally, the lower temperatures African forests tend to experience—as many are situated at slightly higher altitude than forests in Amazonia—could result in limited species tolerace of high temperatures. African forests are also much less species rich than forests in Amazonia and Asia (2, 24), with a relative lack of species in high-temperature African forests (25), and this lower diversity could conceivably drive lower resistance to climate anomalies (26).

Alternatively, the relatively dry conditions of African tropical forests may, perhaps counterintuitively, confer drought resistance. African climate has oscillated between wetter conditions in interglacial periods and cooler and drier conditions in glacial periods (27), so the African pool of species present today may be more drought tolerant because some of the most mesic-adapted biodiversity has been lost over time (28, 29). Drier African tropical forest tree diversity is similar to that of the Amazon or Asia, but tree diversity does not increase with shorter dry seasons in Africa as it does in Amazonia (25), suggesting that most wet-adapted species have been lost and either the dry-adapted species remained or these lineages have diversified more, potentially conferring drought resistance. Indeed, a 40-y drought in West Africa led to an increased abundance of deciduous species in tropical forests in Ghana (30, 31). The relatively cool conditions of African tropical forests might also imply resistance as these forests are further from a potential high-temperature threshold that may limit photosynthesis. Overall, African tropical forests could plausibly be more or less vulnerable to temperature and drought anomalies than Amazonian tropical forests.

Understanding how intact African forests respond to climate anomalies is vital, not least because they have been providing a substantial long-term carbon sink, reducing the rate and magnitude of climate change (5, 6). The impacts of environmental change on African tropical forests are also important because of unique aspects of their structure. African forests typically have high aboveground biomass and so, high carbon storage per unit area—on average, one-third more than Amazon forests (2, 32, 33). African forests are composed of a smaller number of stems, ∼425 ha^−1^ (≥100-mm diameter), compared with ∼600 ha^−1^ in Amazonia and Asia (32) and so, are more dominated by large trees. Hence, even small decreases in growth of the large dominant trees or modest increases in the mortality of these trees could lead to large carbon stock reductions and a loss of the live biomass carbon sink.

The 2015–2016 El Niño event provided a first opportunity to assess the impact of high temperatures and strong water deficits on the ∼450 Mha (6) of African tropical forests. While three very strong El Niño events have occurred in the last 50 y (1982 to 1983, 1997 to 1998, and 2015 to 2016), only the latter occurred after a network of long-term inventory plots had been established in Africa and was poised to capture an El Niño event (5). At the onset of the 2015–2016 El Niño, we organized a specific “emergency” six-nation remeasurement program to capture the impact of the climate anomaly on African tropical forests. We therefore combine climate data with measurements from 100 African Tropical Rainforest Observatory Network (AfriTRON) long-term inventory plots that were remeasured to capture the 2015–2016 El Niño event to address the following questions. 1) Did African tropical forests experience unprecedented temperature anomalies in the 2015–2016 El Niño? 2) Did African tropical forests experience unprecedented drought in the 2015–2016 El Niño? 3) Which climate anomalies drove forest responses to the 2015–2016 El Niño? 4) What were the overall impacts on the monitored old-growth structurally intact tropical forests?

## Methods

### Climate Analysis.

We define the climate of the 2015–2016 El Niño Southern Oscillation event as the 12-mo period from May 2015 to April 2016. These 12 mo capture the two dry seasons per year African forests typically experience (June to August and January to March) and include peak temperatures, which started in March 2015, as land surface temperature anomalies in Africa lagged behind sea surface temperature anomalies that began in November 2014 (34) by 4 mo (22). These 12 consecutive months are also those with the greatest sea surface temperature anomalies (34). We use the same May to April 12 consecutive months for the 1982–1983 and the 1997–1998 El Niño events. To estimate the El Niño climate of African tropical forests, we define “tropical forest” as the Tropical and Subtropical Moist Broadleaf Forest Biome from the WWF Terrestrial Ecoregions of the World map (35). We restrict our analyses to mainland Africa.

### Temperature, Precipitation, and Drought Estimation.

We compare each of the 1982–1983, 1997–1998, and 2015–2016 El Niño events with the climate of the prior decade over African forests. This requires a continuous record from the early 1970s to 2017. We use mean monthly temperatures (0.25° resolution) from the ECMWF Re-Analysis-Interim (ERA-I) dataset for dates from 1979 to 2017 (36). For the years 1970 to 1978, monthly temperature is extracted from the 0.5°-resolution Climatic Research Unit (CRU) ts.4.01 dataset (37). The CRU dataset was resampled to match the resolution of ERA-I and harmonized to Celsius units. ERA-I and CRU are correlated for each month for the overlapping time period (1979 to 2016; i.e., January CRU vs. January ERA-I) for all African tropical forest pixels, and the fit is used to correct the CRU data to match ERA-I (monthly correction coefficients) (*SI Appendix*, Fig. S1). The 1970 to 2017 temperature record (*T*) includes the monthly adjusted CRU data from 1970 to 1978 and ERA-I monthly records from 1979 to 2017.

We also compare each El Niño with the prior decade for only the monitored plot locations by downscaling our climate data to 1 km^2^ using WorldClim Version 2 (38). We downscale by resampling the 1970 to 2017 temperature record to match the resolution of WorldClim. We then use the static 1970 to 2000 WorldClim temperature to correct the 1970 to 2017 record for each plot location by calculating the mean monthly temperature (μ) for the CRU–ERA-I record for the period 1970 to 2000. The monthly difference (*T*_*diff*_ = *T*_μ_ − *T*_*WorldClim*_) of the mean climate *T*_*diff*_ is then used to create a plot-level monthly temperature 1970 to 2017: *T*_*plot*_ = *T* – *T*_*diff*_. Temperature values were then additionally adjusted for any difference in altitude between the plot and the altitude of the 1-km grid cell used for WorldClim interpolation using a lapse rate, so that *T*_*plotalt*_ = *T*_*plot*_ + 0.005 × (*A*_*WorldClim*_ – *A*_*plot*_), where *T* is temperature (degrees Celsius) and *A* is altitude (meters above sea level).

Similarly, continuous precipitation records are required from 1970 to 2017. We use 0.25°-resolution data from the Tropical Rainfall Measurement Mission (TRMM; product 3B43 V7) from 1998 to 2017 (39). Prior to 1998, monthly rainfall is extracted from the Global Precipitation Climatology Centre (GPCC) database [0.5° resolution; Version 7 (40)], chosen as it has more African weather stations than CRU (41). The GPCC dataset is regridded to match the resolution of TRMM. TRMM and GPCC were then correlated for the overlapping time period (1998 to 2003; i.e., January TRMM vs. January GPCC) for all African tropical forest pixels, and the fit is used to correct the GPCC data to match TRMM (monthly correction coefficients) (*SI Appendix*, Fig. S2). Hence, the precipitation record (*P*) includes data from TRMM from 1998 to 2017 and prior to this, adjusted GPCC data.

Precipitation data extraction for plot locations followed a procedure similar to that used for temperature: downscaling to 1-km^2^ resolution using WorldClim [Version 2 (38)]. The GPCC–TRMM precipitation record is resampled to match the resolution of WorldClim, and the mean (μ) GPCC–TRMM precipitation for the period 1970 to 2000 is calculated for each month. As TRMM is known to overestimate precipitation in the driest months and underestimate high-rainfall events (42), the monthly ratio (*P*_*ratio*_ = *P*_μ_/*P*_*WorldClim*_) of the mean precipitation is calculated, and *P*_*ratio*_ is used to adjust monthly precipitation 1970 to 2017: *P*_*plot*_ = *P* × *P*_*ratio*_.

The drought intensity experienced by plots was estimated as the maximum cumulative water deficit (MCWD). The MCWD is calculated as in ref. 42:

$$\text{if}WD_{n-1}–ET_{n}+P_{n}<0,$$

$$\text{then}WD_{n}=WD_{n-1}–ET_{n}+P_{n};$$

$$\text{else}WD_{n}=0,$$

where *WD* = water deficit, *n* = month, *ET* = evapotranspiration, and *P* = precipitation.

We assume a constant monthly evapotranspiration of 100 mm mo^−1^ as has been used in previous studies (6, 9). This is similar to the limited measurements estimating evapotranspiration from forests in West Africa (summarized in ref. 43) and the mean value derived from a data-driven *ET* product for a similar African plot network (6). It allows MCWD to represent a precipitation-driven dry season deficit, meaning that MCWD is temperature independent, allowing us to discriminate between temperature or drought driving changes in growth and mortality. Additionally, we include a variable *ET* estimate based on monthly precipitation and temperature (44) to estimate MCWD, which gives a plot mean *ET* of 101 mm mo^−1^.

For the pre-El Niño monitoring period of a plot, we calculate the mean of the annual MCWD values (i.e., the mean pre-El Niño drought intensity). For the 2015–2016 El Niño census interval, we select the maximum annual MCWD value, as we are interested in the most extreme climate conditions within the El Niño sampling interval for each plot.

### Plot Data Collection and Analysis.

One hundred long-term tropical forest inventory plots in six African countries, within AfriTRON, are included in this study. Of the 244 long-term AfriTRON inventory plots with two censuses prior to 2015 (6), we selected 100 to be recensused based on plots 1) having been recensused shortly before the 2015–2016 El Niño event began, 2) being accessible to recensus after the El Niño finished, and 3) being widely distributed across the continent, all criteria similar to previous sampling of long-term plot networks in the Amazon capturing short-term climate anomalies (9, 10). These permanent sample plots are located in lowland (<800 m above sea level), closed canopy, old-growth, structurally intact tropical forests. All plots have been inventoried at least twice prior to the 2015–2016 El Niño event and once afterward (range from October 2016 to March 2017). The 100 plots are in 26 distinct clusters across six countries: Cameroon, Democratic Republic of the Congo, Gabon, Ghana, Liberia, and Republic of the Congo. The plots were established between 1979 and 2012, but we include only censuses from 2000 onward in the main analysis to avoid potential impacts of the 1982–1983 and the 1997–1998 El Niño events, and to reduce heterogeneity within the dataset. Median plot size is 1 ha, and mean is 0.90 ha (range from 0.2 to 1.1 ha); mean initial census is May 2008, mean pre-El Niño census is April 2014, and mean post-El Niño census is February 2017. The mean monitoring length pre-El Niño was 8.3 y, and the mean length of the El Niño interval was 2.7 y. Data are curated at https://www.forestplots.net/ (45, 46) (Version 2019.1 downloaded on 19 March 2019). *SI Appendix*, Table S1 shows plot details.

In each plot, all trees ≥100 mm diameter are measured, tagged with a unique identifier, and identified to species, where possible. Tree diameter was measured at 1.3 m along the stem from the ground or above buttresses, if present, using standardized methods for all plots (47). In some cases, the point of diameter measurement (POM) had to be moved due to upward growth of buttresses or deformities. For these trees we calculated a single common estimate of growth before and after the POM change, a commonly used approach (5, 6, 8, 19, 48). Stems that reached a diameter ≥100 mm during the census interval were recorded as new recruits.

Field data were checked against rules to identify potential errors, identically for all 100 plots, consistent with previous large-scale analyses (6–8, 32). We assessed trees that increased in diameter >40 mm y^−1^ or shrank >5 mm over an interval to determine if they may have been inaccurately measured in the field. For example, fast-growing species in a canopy gap could grow >40 mm y^−1^, or a rotten trunk could shrink >5 mm in an interval; however, for those deemed potentially inaccurate, the diameter was either interpolated or extrapolated using known measurements from the same stem from other censuses (0.03% of all measurements). When only one accurate measurement was available, growth was estimated by applying the mean growth rate (for diameter classes from 100 to 199 mm and from 200 to 399 mm) or median growth rate for size classes with few stems (for diameters 400+ mm; 0.4% of all measurements across all size classes).

We estimate tree aboveground mass using the allometric equation (49)

$$\text{AGB}=\text{0}.0673\text{×}{\left(\text{ρ}D^{2}H\right)}^{\text{0}.976},$$

where ρ is stem wood density (g cm^−3^), *D* is stem diameter (cm) at 1.3 m or above buttresses, and *H* is height (m). We estimate the aboveground mass of palms using the equation in ref. 50. Wood density measurements for the tree biomass equation were compiled for 730 African species from 608 published sources, they were mostly sourced from the Global Wood Density Database on the Dryad digital repository (https://datadryad.org/stash) (51, 52), and each individual stem in a plot was matched to a species-specific mean wood density value, where possible. Species in both the tree inventory and wood density databases were standardized for orthography and synonymy using the African Plants Database (https://www.ville-ge.ch/musinfo/bd/cjb/africa/index.php?langue=an) to maximize matches (5). For incompletely identified individuals or individuals belonging to species not in the wood density database, we use the mean wood density value for genus if available and then family. For unidentified individuals, we used the mean wood density value of all individual trees in the plot (5, 45).

Tree heights were measured in 93 plots: typically, the 10 largest trees and 10 trees in each of the diameter classes 100 to 199, 200 to 299, 300 to 399, 400 to 499, and 500 to 599 mm were measured, with trees selected only when the top was visible (53). We fit three-parameter regional height–diameter Weibull equations using the local.heights function of the BiomasaFP R package (54). The regions are West Africa (upper Guinea), West Central Africa (western Congo), and East Central Africa (eastern Congo) (33). The parameters (*SI Appendix*, Table S2) were used to estimate tree height from tree diameter for all stems for input into the allometric equation.

We estimate the aboveground biomass in live stems (AGB) in megagrams dry mass per hectare, at each census of each plot; the additions of biomass to each plot over the census interval, as aboveground woody productivity (AGWP), were in megagrams dry mass per hectare per year, and the losses of AGB from the plot, termed AGB mortality, were also in megagrams dry mass per hectare per year. Plot-level carbon gains and losses are increasingly underestimated as census length increases; therefore, to avoid any census interval bias, we corrected for this using the Kohyama method (55). We thus accounted for the carbon additions from trees that recruited and then died within the same interval (unobserved recruitment) and the carbon additions from trees that grew before they died within an interval (unobserved growth). Carbon losses are affected by similar processes, where we add the estimated growth prior to tree death within the interval (unobserved growth) and add the deaths of stems that were newly recruited within the interval (unobserved mortality).

We calculate an analogous set of parameters to AGB carbon gains and carbon losses on a stems basis. We estimate stem density (the number of stems per hectare at a census), stem recruitment (the number of new stems added in a census interval), and stem mortality (the number of stems lost in a census interval). Again, to avoid census interval effects, we estimated stem recruitment and stem mortality following Kohyama et al. (55), accounting for the trees that recruited (unobserved recruitment) and then died (unobserved mortality) within the same interval.

We use the BiomasaFP R package to calculate AGB, AGWP, AGB mortality, stem density, stem recruitment, and stem mortality, including the calculation of the census interval corrections. Pre-El Niño means of these variables are time weighted using the census interval lengths. We express AGB, AGWP, and AGB mortality in carbon terms (net change in carbon stocks, carbon gains, and carbon losses) using the mean carbon fraction of aboveground biomass for tropical angiosperms, 45.6% (56). The differences between the pre-El Niño monitoring period and the El Niño census interval are ∆ net carbon, ∆ carbon gains, and ∆ carbon losses, and for stems, they are ∆ net stems, ∆ recruitment, and ∆ mortality for each plot.

We weight the plots when testing the impacts of climate (temperature, MCWD) on biomass carbon (Δ net carbon, Δ carbon gains, Δ carbon losses) and stems (Δ net stems, Δ recruitment, Δ mortality) using linear regression (Figs. 2 and 3) because larger plots and those monitored for longer provide better estimates of changes in carbon, carbon gains, carbon losses, stems, recruitment, and stem mortality. We calculate an empirical optimum weighting using plot area and pre-El Niño monitoring length by assuming a priori that there is no pattern in the change in carbon, carbon gains, carbon losses, stems, recruitment, or stem mortality with monitoring period or plot size and assess the patterns in the residuals of sampling effort vs. change in carbon, carbon gains, carbon losses, stems, recruitment, or stem mortality, following different weightings, to remove any pattern in the residuals (5). If both an area and monitoring length weight are included, we subtract one to avoid double counting. Weights that remove patterns in the residuals were Δ net carbon, Monitoring length^1/6^ + Area^1/8^ − 1; Δ carbon gains, Monitoring length^1/4^; Δ carbon losses, Monitoring length^1/3^; Δ net stems, Monitoring length^1/2^ + Area^1/4^ − 1; and no weighting for both recruitment and stem mortality. As most plots are relatively similar in size and census monitoring length, the weighting does not affect the results (*SI Appendix*, Table S6).

We test the impacts of climate (temperature, MCWD, and their interaction) on biomass carbon (Δ net carbon, Δ carbon gains, Δ carbon losses) and stems (Δ net stems, Δ recruitment, Δ mortality) using multiple linear regression. We include pre-El Niño climate in models to test whether plots that were already hotter (pre-El Niño temperature) or plots that were already drier (pre-El Niño MCWD) were more or less resistant to environmental change (trees in hotter or drier pre-El Niño climates may contain more hot- or dry-adapted species but also may be closer to physiological temperature or moisture thresholds). We include interactions in our models as we might expect greater impacts in locations that are hotter pre-El Niño and experience a greater temperature anomaly or locations that are drier pre-El Niño and experience a greater MCWD anomaly; plus, high temperatures may exacerbate water deficits. Variables were standardized to allow effect size comparisons. All possible combinations of effect terms were calculated and then restricted to a 95% confidence set (Akaike information criterion [AIC] weights of models sum to 0.95), thereby excluding highly unlikely models. We then model averaged the coefficients of terms (using the AIC weights of each model), meaning that terms with limited support exhibit shrinkage toward zero (57). This multimodel inference was performed using the dredge and model.avg functions of the MuMIn R package (58).

Finally, we test whether the results are robust to the methodological choices necessary to analyze the data. We repeat the main analyses with alternatives; we do not weight the plot data based on plot size and pre-El Niño census monitoring length; we extend the pre-El Niño censuses back to 1984; we test our assumptions associated with the MCWD drought metric; we use an alternative census interval correction procedure; we use an alternative R package, BIOMASS (59), to estimate carbon stocks and plot-level uncertainty; we avoid all allometric assumptions by reanalyzing the results using basal area (changes in the summed cross-sectional area of trees) as the metric rather than biomass carbon; and finally, we exclude the four plots that cooled during the El Niño census interval (this location warmed during the El Niño year but was anomalously cool afterward).

## Results

### Climate.

**Fig. 1.**
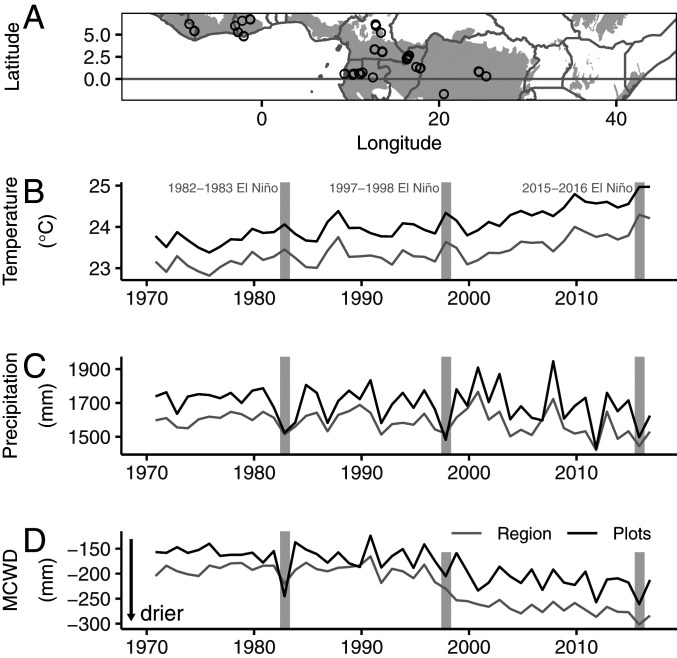
Plot locations (black circles) within the African tropical forest region (gray shading; *A*), annual temperature (*B*), annual precipitation (*C*), and annual maximum drought intensity as MCWD (*D*) for the African tropical forest region (gray lines), and the 100 plot locations used in the study (black lines). The horizontal line in *A* indicates the equator, and gray shading delimits our tropical forest region, using the Tropical and Subtropical Moist Broadleaf Forest Biome from the WWF Terrestrial Ecoregions of the World (35). In *B*–*D* annual means are for the El Niño year May to April for 0.25° grid cells within our tropical forest region. The 100 plots are located in thirty 0.25° grid cells. Gray vertical shading highlights the three very strong El Niño events that have occurred over the past 50 y.

Our 50-y climate record shows that the long-term climate trends across African tropical forests are rising temperatures, decreasing precipitation, and stronger seasonal moisture deficit (Fig. 1). The three very strong El Niño events over the past 50 y are superimposed on these trends (Fig. 1). Consequently, the 1982–1983, 1997–1998, and 2015–2016 El Niño events each increased temperatures by 0.3 °C, 0.3 °C, and 0.6 °C, respectively, over our May to April El Niño year, compared with the temperature of the prior decade, with some evidence that the 2015–2016 anomaly was larger than the previous two strong El Niño events (Table 1). Given the rising temperatures, the mean non-El Niño temperature in the 2010s is greater than the peak temperature during both the 1982–1983 and the 1997–1998 El Niño events (Fig. 1). Thus, in the 2010s, African forests experienced temperatures higher than during the strongest El Niño events of the past, leading to record temperatures in 2015–2016 (Fig. 1*B*).

**Table 1.** Climate anomalies of the last three very strong El Niño events over the past 50 y for the African tropical forest region, mean and 95% confidence interval

El Niño	Mean annual temperature (°C)	Anomaly: decade	Anomaly: 1980–2010	Annual precipitation (mm)	Anomaly: decade	Anomaly: 1980–2010	MCWD (mm)	Anomaly: decade	Anomaly: 1980–2010
1982–1983	23.5 ± 0.02	0.33 ± 0.02	0.08 ± 0.02	1,513 ± 7	−95 ± 10	−84 ± 11	−220 ± 1	−31 ± 1	2 ± 2
1997–1998	23.6 ± 0.02	0.32 ± 0.02	0.26 ± 0.02	1,520 ± 8	−83 ± 11	−78 ± 12	−230 ± 1	−37 ± 2	−8 ± 2
2015–2016	24.3 ± 0.02	0.57 ± 0.02	0.92 ± 0.02	1,444 ± 9	−109 ± 13	−153 ± 12	−302 ± 1	−31 ± 2	−79 ± 2

Across the African tropical forest region, the three very strong El Niño events also decreased precipitation, by ∼100 mm y^−1^, compared with the prior decade, and increased seasonal drought intensity, measured as a more negative MCWD, by ∼30 mm y^−1^ compared to the previous decade (Fig. 1 *C* and *D* and Table 1). These impacts are superimposed on the long-term trends of modestly declining precipitation and a stronger increase in seasonal drought intensity, measured as a more negative MCWD, with some evidence that the 2015–2016 event was the most extreme, particularly for the plot locations (Fig. 1 *C* and *D* and Table 1). Overall, the precipitation changes were less extreme than the changes in seasonal drought (Fig. 1 and Table 1).

In the 2015–2016 El Niño, the 100 AfriTRON plots experienced record mean annual temperatures of 25.0 °C ± 0.03 °C (95% CI), low total annual precipitation (average 1,498 ± 24 mm), and a record low MCWD, with mean −261 ± 2 mm (April to May year) (Fig. 1 and Table 1). Comparing the plot census interval that captures the 2015–2016 El Niño with the plot pre-El Niño census period, 96 of the 100 plots had higher mean monthly temperature during the El Niño plot census interval (mean +0.28 °C ± 0.04 °C). All 100 plots had more negative MCWD (mean −97 ± 12 mm), and 67 plots also had lower total annual precipitation (mean −38 ± 32 mm y^−1^). These anomalies are smaller than those for the 12-mo El Niño year because the plot El Niño mean census length was 2.7 y, so the climate anomaly is diluted by the inclusion of months of more usual conditions. The 100 monitored plot locations are hotter, wetter, and less droughted than the region as a whole because the wider region also includes currently degraded fringes of the biome, but both the plots and region have similar trends of increasing temperature, decreasing rainfall, and stronger seasonal drought (Fig. 1).

### Drivers of Biomass Carbon Dynamics.

**Fig. 2.**
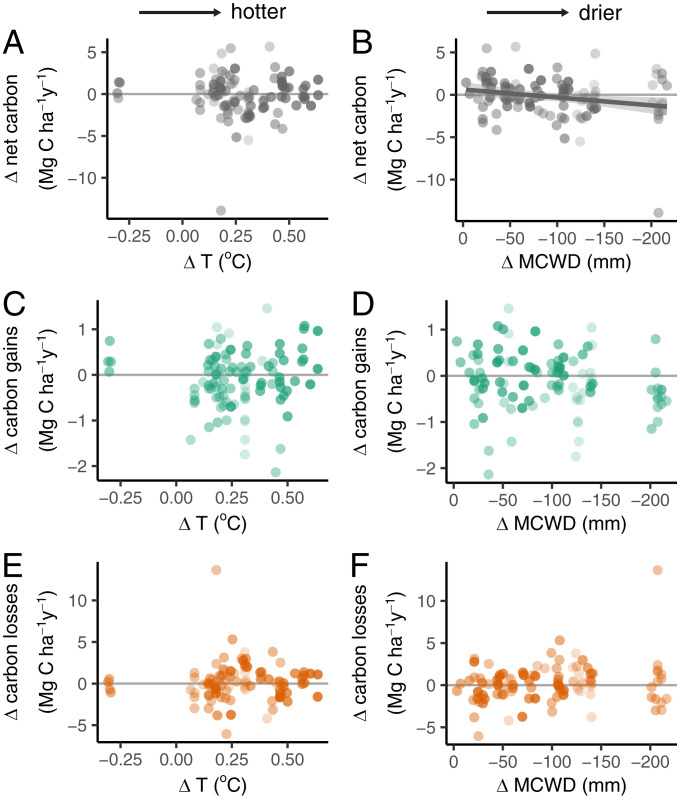
Temperature (*A*, *C*, and *E*) and drought (*B*, *D*, and *F*) impacts on aboveground biomass carbon dynamics of 100 long-term forest plots. The net carbon change (gray; *A* and *B*), carbon gains from tree growth and newly recruited stems (green; *C* and *D*), and carbon losses from mortality (orange; *E* and *F*) of the censuses capturing the El Niño event minus the pre-El Niño plot monitoring period for 100 long-term inventory plots. The temperature change, Δ temperature (*T*; *A*, *C*, and *E*), is the mean temperature during the El Niño minus mean temperature pre-El Niño using the plot census dates. The change in drought intensity, Δ MCWD (*B*, *D*, and *F*), is the maximum MCWD during the El Niño minus the mean MCWD pre-El Niño using the plot census dates. Point shading from light to dark denotes greater weighting, empirically derived from plot area and the length the plot was monitored pre-El Niño. The solid line shows the only significant linear model (slope = −0.009; *P* ≤ 0.05; shading is 95% CI).

**Fig. 3.**
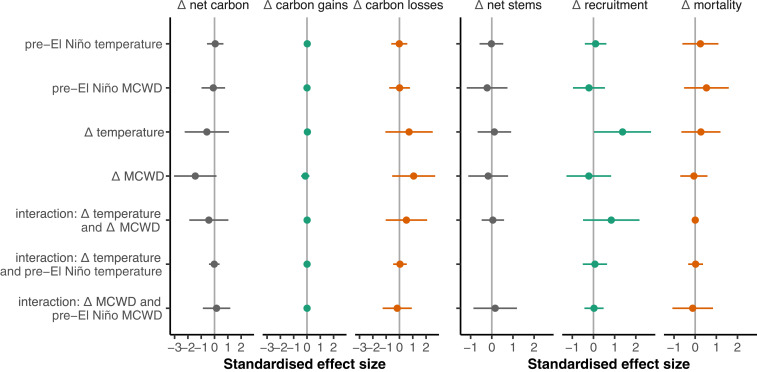
Effect sizes of change in carbon (*Left*) and stems (*Right*) in 100 African tropical forest plots over the 2015–2016 El Niño. Points show coefficients from linear models with multimodel inference, standardized to represent the change in the response variable for one SD change in the explanatory variable. Error bars show 95% CIs. The models explained 11, 5, and 11% of variation in Δ net carbon, Δ carbon gains, and Δ carbon losses, respectively, and 7, 15, and 10% of variation in Δ net stems, Δ recruitment, and Δ mortality, respectively. Coefficient values are in *SI Appendix*, Tables S3 and S4.

The record high temperatures of the 2015–2016 El Niño had no detectable effect on forest carbon gains, losses, or the carbon sink in live biomass over the period of monitoring; higher temperatures were not correlated with carbon gains, losses, or the strength of the live biomass carbon sink (Fig. 2 *A*, *C*, and *E*). Considering drought, forests experiencing stronger MCWD showed a small but nonsignificant reduction in carbon gains (Fig. 2*D*) and a larger but also nonsignificant increase in carbon losses (Fig. 2*F*), leading to significantly greater reductions in the live biomass carbon sink in more strongly droughted plots (*P* ≤ 0.05) (Fig. 2*B*). Thus, relative to pre-El Niño, forests subjected to a 100-mm increase in MCWD water deficit lost 0.3 Mg C ha^−1^ y^−1^, dominated by carbon losses. Multimodel inference, including Δ temperature and Δ MCWD, confirms that Δ MCWD is more important than Δ temperature in determining El Niño carbon dynamics responses (Fig. 3). Drought, not temperature, drives the biomass changes seen in plots.

The lack of a negative temperature response and the modest negative drought response are both robust to the various methodological choices necessary to analyze the data, summarized in *SI Appendix*, Table S6. The responses of carbon gains, carbon losses, and the net carbon sink in live biomass remain very similar if we do not weight the plots by size and pre-El Niño monitoring length (*SI Appendix*, Fig. S6), include a longer pre-El Niño plot monitoring length back to 1984 (*SI Appendix*, Fig. S7), employ an alternative census interval correction procedure (*SI Appendix*, Fig. S8), use different drought metrics (*SI Appendix*, Figs. S9 and S10), use an alternative R package to analyze changes in carbon stocks (*SI Appendix*, Fig. S11), or remove potential allometric uncertainties by analyzing changes in basal area (*SI Appendix*, Figs. S12 and S13). In these cases, the slopes of the temperature relationships remain very similar and are not significant (*SI Appendix*, Table S6). However, for the temperature anomaly–carbon gains relationship, if we exclude four plots that cooled during their El Niño census interval (code MDJ; they warmed during our May to April El Niño year but were anomalously cool afterward), then the temperature–carbon gains linear model has a significant positive slope (*P* = 0.045) (*SI Appendix*, Fig. S14*B* and Table S6), although when our multimodel inference excluding the same four plots is repeated, the positive temperature–carbon gains effect is nonsignificant (*P* = 0.1) (*SI Appendix*, Fig. S15). In the case of MCWD, again slopes remain very similar and relationships are always at the margins of significance under the various methodological choices, with stronger drought decreasing carbon gains and increasing carbon losses (*SI Appendix*, Figs. S6–S13 and Table S6). Calculating MCWD using variable evapotranspiration (which includes temperature) does not alter the slope of the relationships, but it improves significance of the relationship with carbon gains and losses (*SI Appendix*, Fig. S9 and Table S6), further suggesting that drought impacted African forests but only modestly.

### El Niño Impact on Biomass Carbon Dynamics.

**Fig. 4.**
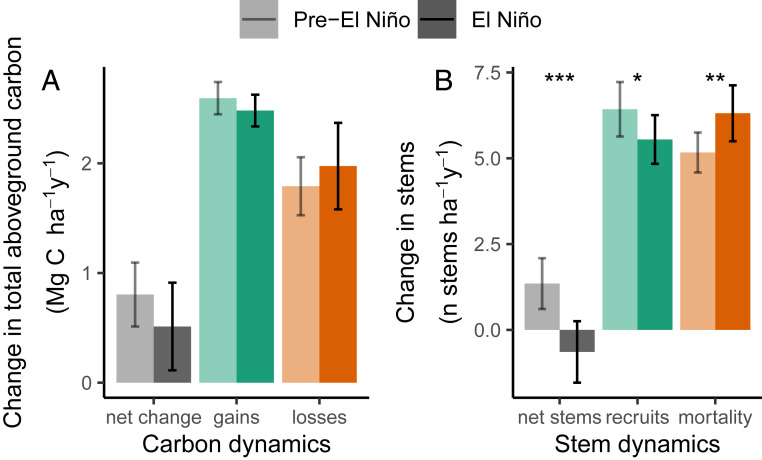
Carbon (*A*) and stem dynamics (*B*) as net change, gains, and losses, for pre-El Niño and during the 2015–2016 El Niño (lighter and darker shading, respectively) for 100 long-term forest plots (mean and 95% CI). Significant differences are defined by paired *t* tests. *P ≤ 0.05; **P < 0.01; ***P < 0.001.

Plot-level carbon dynamics over the 2015–2016 El Niño were similar to those pre-El Niño. Carbon gains from tree growth and the recruitment of new trees were 4.4% lower in the interval spanning the 2015–2016 El Niño (mean 2014.3 to 2017.1) compared with the pre-El Niño censuses (mean 2008.4 to 2014.3), a marginally significant difference (2.59 ± 0.15 to 2.48 ± 0.14 Mg C ha^−1^ y^−1^; paired *t* test, *P* = 0.07) (Fig. 4*A*). Excluding allometric uncertainty by calculating basal area gains increased the reduction in tree growth and new tree recruitment to 5.5%, a significant decrease (*P* = 0.03) (*SI Appendix*, Fig. S13). Growth responses by size class indicate that small trees grew less during the El Niño census interval. Small trees had significantly lower median growth rates in the El Niño compared with the pre-El Niño measurement period (trees 100- to 199-mm diameter had a growth rate decrease of 0.15 ± 0.11 mm y^−1^ from 0.83 to 0.68 mm y^−1^; *P* < 0.01), as did the medium size-class trees (trees 200- to 399-mm diameter had a decrease of 0.17 ± 0.14 mm y^−1^ from 1.64 to 1.47 mm y^−1^; *P* ≤ 0.05). The median growth rate of large trees (400+-mm diameter) was also lower but did not significantly decline (0.14 ± 0.21 from 2.86 to 2.72 mm y^−1^; *P* = 0.2). Hence, the growth of smaller trees appeared to be more negatively impacted by the El Niño conditions than the growth of large trees.

There was a nonsignificant 10% increase in carbon losses from mortality in the El Niño compared with pre-El Niño censuses (1.79 ± 0.26 to 1.97 ± 0.39 Mg C ha^−1^ y^−1^; paired *t* test, *P* = 0.4) (Fig. 4*A*). Excluding allometric uncertainty by calculating basal area losses showed an increase of 12%, a nonsignificant increase (*P* = 0.30) (*SI Appendix*, Fig. S13).

Both the overall modest nonsignificant decrease in carbon gains and modest nonsignificant increase in carbon losses during the El Niño census interval (Fig. 4*A*) combined to reduce the live biomass carbon sink by an average of 36%; however, this was also a nonsignificant reduction, from 0.80 ± 0.29 to 0.51 ± 0.40 Mg C ha^−1^ y^−1^ (paired *t* test, *P* = 0.2) (Fig. 4*A*). Excluding allometric uncertainty by calculating the net change in basal area showed a decrease of 49%, a nonsignificant reduction (*P* = 0.11) (*SI Appendix*, Fig. S13). Despite the extreme conditions, the 100 plots remained a significant net aboveground live biomass carbon sink over the census interval that included the 2015–2016 El Niño, at 0.51 ± 0.40 Mg C ha^−1^ y^−1^. The El Niño had modest impacts on the overall carbon dynamics of the 100 plots studied. While drought significantly impacted the aboveground live biomass carbon sink in plots (Fig. 2), not enough plots were droughted severely enough for that trend to drive the mean sink strength across the 100 plots down to zero or to reverse it (Fig. 4).

### Stem Dynamics.

An analysis of stem dynamics, rather than biomass carbon dynamics, and the potential drivers of change shows, surprisingly, stem recruitment increasing with increasing temperature, and as expected decreasing with more negative MCWD (*SI Appendix*, Fig. S3). Indeed, recruitment is the process most responsive to the climate anomaly, responding positively to temperature and negatively to drought (*SI Appendix*, Fig. S3). The increase in stem recruitment with temperature could potentially have been driven by recruitment of low wood density stems, which might have benefited from more light reaching the understory, but there is no evidence of this as the median wood density of recruits did not change (0.63 g cm^−3^ pre-during the El Niño, 0.62 g cm^−3^ during the El Niño; paired *t* test, *P* = 0.4). Somewhat surprisingly, stem mortality showed no significant relationship with either temperature or drought anomalies (*SI Appendix*, Fig. S3).

Considering the 100 plots together, there was a significant decrease in stem recruitment during the El Niño event census interval by 14% (from 6.4 ± 0.8 to 5.5 ± 0.7 stems ha^−1^ y^−1^; paired *t* test, *P* ≤ 0.05) (Fig. 4*B*). Stem mortality significantly increased by 22% (from 5.2 ± 0.6 to 6.3 ± 0.8 stems ha^−1^ y^−1^; *P* < 0.01) (Fig. 4*B*). Together, these led to a switch from an increase of 1.3 ± 0.7 stems ha^−1^ y^−1^ pre-El Niño to decline of 0.6 ± 0.9 stems ha^−1^ y^−1^, a significant decrease in stem density during the El Niño census interval (*P* < 0.001) (Fig. 4*B*).

The significant increase in stem mortality without simultaneous significant increases in carbon losses and the larger increase in stem mortality than carbon losses from mortality (Fig. 4) implies a loss of smaller trees, which is what we found. Stem mortality rate increased overall (an increase of 0.2 ± 0.2%, from 1.2 to 1.5%; paired *t* test, *P* ≤ 0.05), but this was mostly due to small (100- to 199-mm diameter: 0.3 ± 0.2% from 1.4 to 1.7%; *P* < 0.01) and medium (200- to 399-mm diameter: 0.2 ± 0.2% from 1.1 to 1.3%; *P* ≤ 0.05), rather than large trees (400+-mm diameter: 0.07 ± 0.2% from 1.2 to 1.2%; *P* = 0.7). Furthermore, the median diameter of dying trees decreased significantly from 190-mm diameter pre-El Niño to 174 mm in the El Niño census, a decline of 16 ± 14 mm (*P* ≤ 0.05). When separated into size classes, the median diameter of dying trees in the smallest size class, 100- to 199-mm diameter, decreased by 4 ± 4 mm (135.9 to 131.7 mm; *P* = 0.06), while the median diameter of the other size classes did not change (200- to 399-mm diameter: 272 to 273 mm; *P* = 0.8; 400+-mm diameter: 584 to 578 mm; *P* = 0.8). The median size of surviving trees overall also does not change (179- to 179-mm diameter; *P* = 0.9), and there is no change in the median wood density of dying (0.64 to 0.63 g cm^−3^; *P* = 0.6) nor surviving trees (0.65 to 0.65 g cm^−3^; *P* = 0.7). Overall, stem mortality increased significantly over the El Niño census interval, and it was predominantly smaller trees that died.

## Discussion

African tropical forests experienced record heat and drought in the 2015–2016 El Niño, yet the 100 inventory plots monitored through this extreme climate anomaly maintained net biomass carbon uptake over the 2.7-y period they were measured, at 0.51 ± 0.40 Mg C ha^−1^ y^−1^. Maintenance of the live biomass carbon sink suggests that African forests were resistant to this short-term extreme climate anomaly, albeit that the carbon sink was maintained at a lower rate. Alternatively, it is theoretically possible that impacts were more substantial but we failed to detect them, perhaps due to limited sampling or because the main impacts of the El Niño are delayed beyond the time of the plot remeasurement, and so the impact is ultimately larger than we estimate.

Our plot sampling is likely to be sufficient to detect a shutdown of the sink if it occurred because our sample matches or exceeds the sample remeasured to capture the 2005 Amazon drought [Phillips et al. (9): 55 plots] and the 2010 Amazon drought [Feldpausch et al. (10): 97 plots] and greatly exceeds the sample used for the 1997–1998 El Niño in Bornean forests [Qie et al. (8): 19 plots], all of which detected that the live biomass carbon sinks over large tropical forest regions temporarily shut down or reversed with drought. In particular, our emergency plot recensus campaigns to capture the climate anomaly, field methods, data processing, and analysis were designed to be comparable with the past Amazonian analyses, which showed a sink shutdown. Reanalyses of our data using alternative approaches did not alter the results, with slopes of relationships remaining very similar (*SI Appendix*, Figs. S6–S13 and Table S6). Critically, halting the live biomass carbon sink requires large increases in tree mortality, as seen during the Amazon 2005 and 2010 droughts and Bornean 1997–1998 El Niño event (8–10), or large decreases in carbon gains from tree growth, as seen during the Amazon 2010 drought (10). Neither signature is seen in the African forest response to the 2015–2016 El Niño, which is dominated by modest negative consequences for smaller stems.

It is also unlikely that there was a substantial delayed mortality response to the El Niño that we missed, because most trees die within a few months of tropical drought events (8, 9). Our average El Niño census interval was 2.7 y long, similar to the 2.0-y interval in Phillips et al. (9) and the 3.5-y interval in Qie et al. (8) that did detect both significantly elevated biomass mortality and a carbon sink shutdown in Amazonian forests and Southeast Asian forests, respectively. A protracted response to the El Niño is conceivable, as some extratropical studies show mortality lagging behind drought events (60, 61). However, recent analyses have not shown a lagged response of carbon losses in Africa to drought (6), including no rising carbon losses from mortality after the end of the 1997–1998 El Niño in African forests, although sample sizes were small at that time (6). Additionally, remotely sensed microwave data used to estimate carbon stocks show no large reduction in carbon stocks after the end of the 2015–2016 El Niño, again inconsistent with a delayed mortality response (62, 63). Finally, simulations using land surface model data and atmospheric inversions also do not show a reduction in carbon stocks after the end of the 2015–2016 El Niño in humid African forests, instead they suggest a modest recovery (64). In summary, the available evidence does not support a major lagged tree mortality response to the 2015–2016 El Niño event in humid African forests.

While we lack standardized whole-forest drought and warming experiments across the tropics or ground-based studies of the same drought or high-temperature event on different continents, the data available suggest that humid lowland tropical African forests are, on average, more resistant to short-term extreme climate anomalies when compared with previous observations of Asian (cf. refs. 8 and 65) or Amazonian forests (cf. refs. 9 and 10). This interpretation is consistent with a remotely sensed gross primary productivity decline during the El Niño that was smaller in African humid forests than in Amazonia (64); a decadal-scale analysis that shows African forest carbon gains are less responsive to drought than Amazon forests (6); and evidence that temperatures in African forests are on average ∼1 °C cooler than Amazonia due to being at slightly higher altitude (6), hence farther from any temperature threshold that may impact forest function (19). Independent analyses of long-term inventory data showing pantropical temperature thresholds for reductions in carbon stocks (19) suggest that during the 2015–2016 El Niño, African forests tended to remain below the critical temperature value. Overall, these studies are consistent with our data suggesting greater resistance of African forests to anomalous temperatures and drought compared with Amazonian forests.

It is important to note that the extreme climate anomaly was shorter than the 2.7-y monitoring period. We could assume that the pre-El Niño sink continued until the El Niño event began and returned to this level afterward, suggesting that there was a stronger impact of the anomaly on forest carbon dynamics than is apparent from our dataset. If the pre-El Niño sink occurred at all times except during our 12-mo El Niño year, then the forest trees would have sequestered 0.02 Mg C ha^−1^ y^−1^ during the El Niño (i.e., the sink turned off, but these forests probably did not become a temporary carbon source). If the impacts were concentrated in just the strongest dry season months, then these forests were a short-term source of 0.58 Mg C ha^−1^ y^−1^ for those 3 mo. Under this potential scenario, the immediate return of the forest to a strong sink after being a very temporary strong source could then be interpreted as African tropical forests being resilient rather than resistant to environmental change. However, the lack of strong correlations between the biomass carbon sink and temperature or drought anomalies (Fig. 2) suggests such extreme short-term carbon flux changes in humid African forests are unlikely. Furthermore, if these forests exhibited a large source and then recovered with a large sink, they would show a signature in our plot data of high carbon losses and a large increase in carbon gains over the 2.7-y period, which we did not find (Fig. 4). African tropical forests thus appear resistant rather than resilient to the record heat and drought.

Our plots monitor the stock of carbon in live biomass, which is gross primary productivity minus autotrophic respiration and biomass losses from tree mortality. In addition, there is also the heterotrophic respiration flux of carbon to the atmosphere from necromass (dead trees) and soils. The net of these fluxes is net biome productivity. From an atmospheric perspective, the full impacts of the reduction in the live biomass carbon sink from slowing carbon gains are experienced immediately, but the contribution from rising carbon losses is delayed because dead trees do not decompose instantaneously, meaning that the contribution of biomass losses to the net biome productivity response to the El Niño is also likely to be modest. For intact tropical forest soil carbon, fluxes to the atmosphere are typically lower during drought conditions (66, 67), suggesting that the overall net biome productivity response in African forests is likely to be muted because the small 4.4% decrease in carbon gains from forest growth (i.e., net primary productivity) may be somewhat offset by an accompanying smaller soil carbon flux from decreased heterotrophic respiration. Consistent with this, remotely sensed CO_2_ data, which capture the net flux from all these processes and any land use change emissions, suggest that the regions of tropical forest where our plots are located were an ongoing carbon sink throughout 2015 and 2016 (68). This independent evidence, and the fact that these forests have been a sink over 2.7 y while experiencing unprecedented heat and drought, reinforces our conclusion that African forests were resistant to the 2015–2016 extreme climate anomaly.

### Regional Carbon Implications.

A recent assessment of the long-term carbon sink in African forests estimates it to be 0.46 Pg C y^−1^ (0.37 to 0.56, 95% CIs) for the years 2000 to 2010 (6), and our results—assuming no changes to soils or necromass—suggest the sink reduced by 36% over our El Niño census period; hence, over the 2.7-y period, the sink is estimated as 0.29 Pg C y^−1^ (i.e., 64% of 0.46 Pg C y^−1^). While mindful that the reduced sink captured by plot measurements does not include necromass and soil fluxes and is extrapolated to selectively logged forests (where typically <1 tree ha^−1^ is removed) as well as intact forests, our results are in broad agreement with a recent satellite-derived estimation of the carbon sink in African humid forests (68). Palmer et al. (68) analyzed atmospheric CO_2_ data to show that while Africa as a whole was a large source of carbon to the atmosphere over the El Niño, this was due to carbon release from northern Africa. For the Congo Basin, in particular the western region of the Congo Basin and contiguous forests where the majority of our plots are located, the CO_2_ data show a carbon sink in this region in both 2015 and 2016, consistent with the ongoing net live biomass carbon sink in 2015 and 2016 we find using the plot inventory data.

By contrast, Liu et al. (34), also analyzing satellite-derived atmospheric CO_2_ data but combined it with a vegetation model and came to a different conclusion, showing a source of 0.8 ± 0.2 Pg C y^−1^ in the El Niño of 2015–2016, compared with the 2010–2011 strong La Niña year for the African continent. The result of Liu et al. (34) is due to high-surface temperature anomalies driving an increase in ecosystem respiration, which is difficult to compare with our study because we consider only tropical forests rather than the whole African continent, and our plot data do not measure net biome productivity. However, plant respiration increases in our plot data would manifest as reductions in carbon gains correlated with increased temperature; while our forest biomass carbon gains declined modestly by 4.4%, this was correlated with drought not temperature, and the overall decline was too weak to stop or reverse the live biomass carbon sink in the plots. Alternatively, the flux identified by Liu et al. (34) could come from outside tropical forests or from tropical forest soils. If heterotrophic respiration increases as temperatures rise, then decomposition, net mineralization, and nutrient availability may increase and could stimulate tree growth, as one of our analyses that excluded four plots that cooled suggests (*SI Appendix*, Fig. S14). Hence, increased heterotrophic respiration could potentially contribute to the muted response of carbon gains to the climate anomaly that we see. It should be noted that the Liu et al. (34) respiration term is the residual after accounting for the estimates of the net biome exchange of carbon, gross primary productivity, and fire losses; thus, it is likely to be uncertain and potentially dominated by systematic errors in other terms. Alternatively, the carbon losses in Liu et al. (34) could come from African ecosystems outside the core humid forest areas where our plots are located. Simulations using atmospheric inversions and land use models show such a response with much of the El Niño response in the drier parts of Africa, rather than the wetter parts (64). This is echoed by remotely sensed observations showing that the semiarid parts of Africa dominate the Africa signal of net carbon loss during the El Niño (63). Collectively, these analyses are broadly consistent with our plot results of an approximately one-third reduction in the carbon sink in live biomass of intact forests over the El Niño, but given that satellite-derived microwave and CO_2_ data are collected at larger scales and include the consequences of land use change as well as forest responses to temperature and precipitation anomalies, precise like for like comparisons are difficult.

### Modest African Responses.

Our results suggest that African forests appear to be more resistant to drought than many Amazonian or Bornean forests. Our African data show that the carbon impact of the drought in Africa was a reduction of 0.29 Mg C ha^−1^ y^−1^ in live aboveground biomass (pre-El Niño net carbon sink of 0.80 Mg C ha^−1^ y^−1^, El Niño net carbon sink of 0.51 Mg C ha^−1^ y^−1^) or 0.2% of aboveground carbon stocks given the initial aboveground biomass carbon of 164 Mg C ha^−1^. Droughts in Amazonia had larger impacts. During the 2005 drought (9), they lost 0.73 Mg C ha^−1^ y^−1^ (0.41 Mg C ha^−1^ y^−1^ predrought sink to a loss of 0.32 Mg C ha^−1^ y^−1^ during the drought) or 0.5% of their aboveground biomass carbon assuming aboveground biomass carbon of 140 Mg C ha^−1^ (2). During the 2010 drought (10), they lost 0.81 Mg C ha^−1^ y^−1^ (0.61 Mg C ha^−1^ y^−1^ predrought sink to a loss of 0.20 Mg C ha^−1^ y^−1^ during the drought) or 0.6% of aboveground biomass carbon assuming 140 Mg C ha^−1^ aboveground biomass (2). In Borneo (8), during the 1997–1998 El Niño there was a loss of 1.44 Mg C ha^−1^ y^−1^ (0.52 Mg C ha^−1^ y^−1^ pre-El Niño sink to a loss of 0.94 Mg C ha^−1^ y^−1^ during the El Niño drought) or 0.7% of aboveground biomass carbon assuming 197 Mg C ha^−1^ aboveground biomass (2). Thus, the response of African tropical forests to the 2015–2016 including record drought is less on both an absolute and relative basis than the response measured in forests in Amazonia and Borneo to earlier droughts.

Why then were the El Niño impacts small for African tropical forests? Muted African forest responses to the 2015–2016 El Niño might be expected as some of the strongest water deficit anomalies occurred in the wettest plots (*SI Appendix*, Fig. S4), while peak temperature increases across African tropical forests were relatively short-lived. Nevertheless, these were record conditions regionally, and they failed to cause intact African tropical forests to lose, on average, any live biomass (these forests were, on average, larger at the end of the El Niño census than at the beginning). One reason why African forests may be more drought-adapted than most Amazonian or Southeast Asian forests is due to biogeographic history having favored the persistence, expansion, and perhaps diversification of more drought-adapted species (25, 27), alongside adaptation to the relatively dry contemporary conditions across the continent (22). A recent analysis using inventory data also suggests that African forests are more drought resistant but not more heat resistant than Amazonian forests (6).

With relatively few stems and relatively high biomass per hectare, African tropical forests are more dominated by large trees than forests typically found in Amazonia or Southeast Asia (32). Because of their structure, small trees may be more subordinate in these ecosystems than those in forests in Amazonia or Asia. In Africa during the 2015–2016 El Niño, the response of large dominant trees was variable and not strongly directional, whereas small trees grew less and died more, perhaps due to more limited access to water than the large trees. These negative impacts on small trees show that African forests were negatively impacted by the El Niño, but responses were modest overall because large trees disproportionately influence forest stocks and total biomass (33, 69–71). African forests are structurally unique, possibly due to megafauna maintaining their large trees, and their large, long-lived trees may buffer ecosystem responses to environmental change (32, 69). Thus, it may be the existence of a more complete megafauna that is conferring resistance to record-breaking climate anomalies by encouraging large and long-lived tree species (70)—that may be better able to withstand periods of adverse conditions—to dominate the canopy.

Unlike many droughts elsewhere (65, 72), we documented no increase in large tree (400+-mm diameter) stem mortality rates (pre-El Niño 1.16%, El Niño 1.23%; paired *t* test, *P* = 0.7), which implies that rates of hydraulic failure in African tropical forests during the 2015–2016 El Niño only increased slightly, if at all. The nonsignificant 6% increase in stem mortality for trees with 400+-mm diameter is much smaller than the 70 to 100% increase seen as a result of previous droughts observed in Amazonia and Borneo when considering droughts of similar intensity (65). The lack of a substantial increase in large tree mortality further explains the limited impacts of the El Niño on African forest carbon stocks.

In terms of biomass carbon gains, there was a modest 4.4% decrease in gains over the El Niño census period (*P* = 0.07) (Fig. 4*A*) and a 5.5% decrease when considering gains on a basal area basis (*P* = 0.03) (*SI Appendix*, Fig. S13). The African forest decrease in biomass gains, 0.11 ± 0.12 Mg C ha^−1^ y^−1^, is a more muted response than the Amazonian forest response to the 2010 drought, where gains reduced by 0.50 Mg C ha^−1^ y^−1^ [0.31 to 0.78, 95% CIs; *P* < 0.001 (10)], but is similar to the nonsignificant decrease during the 2005 Amazon drought (9) and the lack of change in carbon gains in Bornean forests during the 1997–1998 El Niño event (8). However, if we consider only those 96 plots that warmed during their El Niño plot census interval, there was a positive relationship between temperature anomaly and carbon gains (*P* ≤ 0.05), suggesting resistance. While an unexpected result, this could occur if higher temperatures increase mineralization rates and nutrient release (73), particularly toward the start of the climate anomaly (66, 67, 74). Alternatively, short-term shifts in allocation patterns may be causing the trend, as studies of Amazonian trees have shown allocation shifts in hot dry seasons (75, 76). Another possibility is high temperatures leading to some canopy leaf loss, resulting in greater carbon gains if the extra light increases photosynthesis through the canopy. Whatever the mechanism, the lack of a strong negative relationship between carbon gains and temperature anomaly during the El Niño climate anomaly suggests that African tropical forests may be more resistant to regionally higher temperatures, consistent with recent cross-continental analyses (19).

In summary, we show that the unprecedented 2015–2016 climate anomaly, including record air temperatures and water deficits, was insufficient to reverse the long-standing net carbon uptake into live biomass in intact forests. This surprising resistance of structurally intact African forests and their continued live biomass carbon sink is in contrast to the measured responses of Amazonian and Bornean forests to droughts using long-term plots (8–10). However, our results are consistent with a recent study of satellite-derived atmospheric CO_2_ concentration data in Africa showing a carbon sink in 2015 and 2016 in the regions where most of the AfriTRON plots are located (68). Resistance of African tropical forests to rapid environmental change might be due to one or more of the following: 1) their structure, as African tropical forests are more dominated by large trees (32), which in turn, may be a result of environmental conditions or the retained megafauna (70); 2) African tree species are more resistant, on average, to environmental change as they have typically tolerated more extreme environmental change in glacial to interglacial cycles than Amazonian or Asian tree species (25); or 3) contemporary environmental conditions in African forests are not as hot nor warming as fast as Amazonian forests (6), and hence, thresholds where resistance is breached have not been crossed yet (19). Our detection of the resistance of African tropical forests to unprecedented climate conditions was only possible because the AfriTRON ground monitoring network was already in place. Further progress will be made in understanding changes in African tropical forests if this network of long-term plots continues to be monitored and is expanded to undersampled areas, coupled with identifying traits that confer temperature and drought resistance and the integration of our on-the-ground plot network with CO_2_ flux measurements, experiments, modeling, and new remote-sensing technologies.

## Supplementary Material

Supplementary File

## Data Availability

The input data and R code are available on ForestPlots (https://doi.org/10.5521/forestplots.net/2021_4) (77).
