# Supplementary material for: Resistance of African tropical forests to an extreme climate anomaly
Source: Proc Natl Acad Sci U S A. 2021 May 17;118(21):e2003169118. doi: 10.1073/pnas.2003169118 (PMC8166131; doi:10.1073/pnas.2003169118)
Supplement: Supplementary File [file pnas.2003169118.sapp.pdf]

PNAS
www.pnas.org

Supplementary Information for
Resistance of African tropical forests to an extreme climate anomaly

Amy C. Bennett , Greta C. Dargie, Aida Cuni-Sanchez , John Tshibamba Mukendi, Wannes Hubau, Jacques M. Mukinzi, Oliver L. Phillips, Yadvinder Malhi, Martin J. P. Sullivan, Stephen Adu Bredu, Kofi Affum-Baffoe, Christian A. Amani, Lindsay F. Banin, Hans Beeckman, Serge K. Begne, Yannick E. Bocko, Pascal Boeckx, Jan Bogaert, Terry Brncic, Eric Chezeaux, Connie J. Clark, Declan L.M. Cooper, Armandu K. Daniels, Thales de Haulleville, Jean-Louis Doucet, Fidèle, Evouna Ondo, Corneille E.N. Ewango, Ted R. Feldpausch, Ernest G. Foli, Christelle Gonmadje, Jefferson S. Hall, Olivier J. Hardy, David J. Harris, Suspense A. Ifo, Kathryn J. Jeffery, Marie-Noël Kamdem Djuikouo, Elizabeth Kearsley, Miguel Leal, Aurora Levesley, Jean-Remy Makana, Faustin Mbayu Lukasu, Vincent P. Medjibe, Vianet Mihindu, Sam Moore, Natacha Nssi Begone, Georgia C. Pickavance, John R. Poulsen, Jan Reitsma, Bonaventure Sonké, Terry C.H. Sunderland, Hermann Taedoumg, Joey Talbot, Darlington S. Tuagben, Peter M. Umunay, Hans Verbeeck, Jason Vleminckx, Lee J.T. White, Hannsjoerg Woell, John T. Woods, Lise Zemagho and Simon L. Lewis

Amy C. Bennett
Email: a.c.bennett@leeds.ac.uk

This PDF file includes:

Figures S1 to S15
Tables S1 to S7
SI References

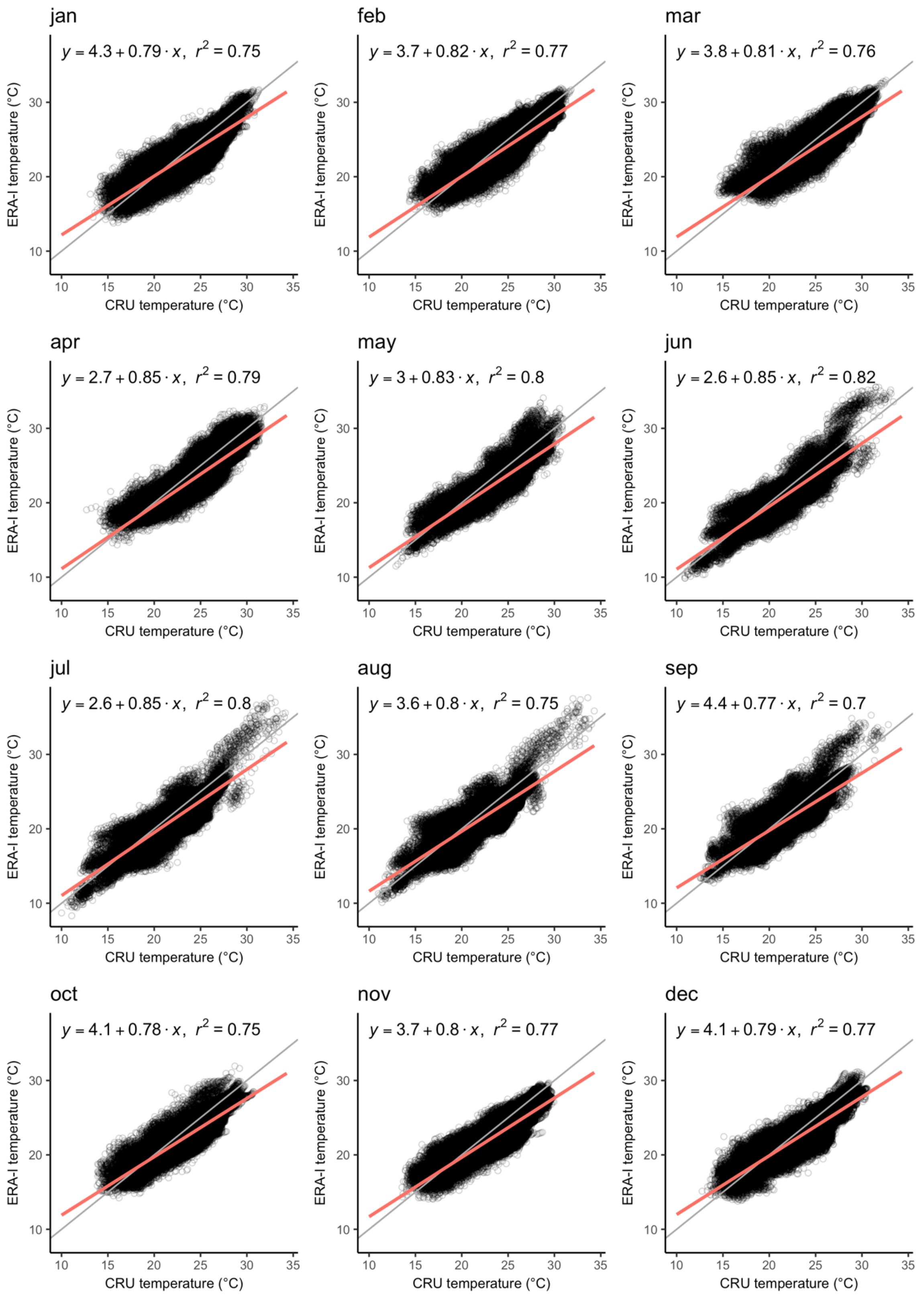

Fig. S1. Monthly correction coefficients for temperature.

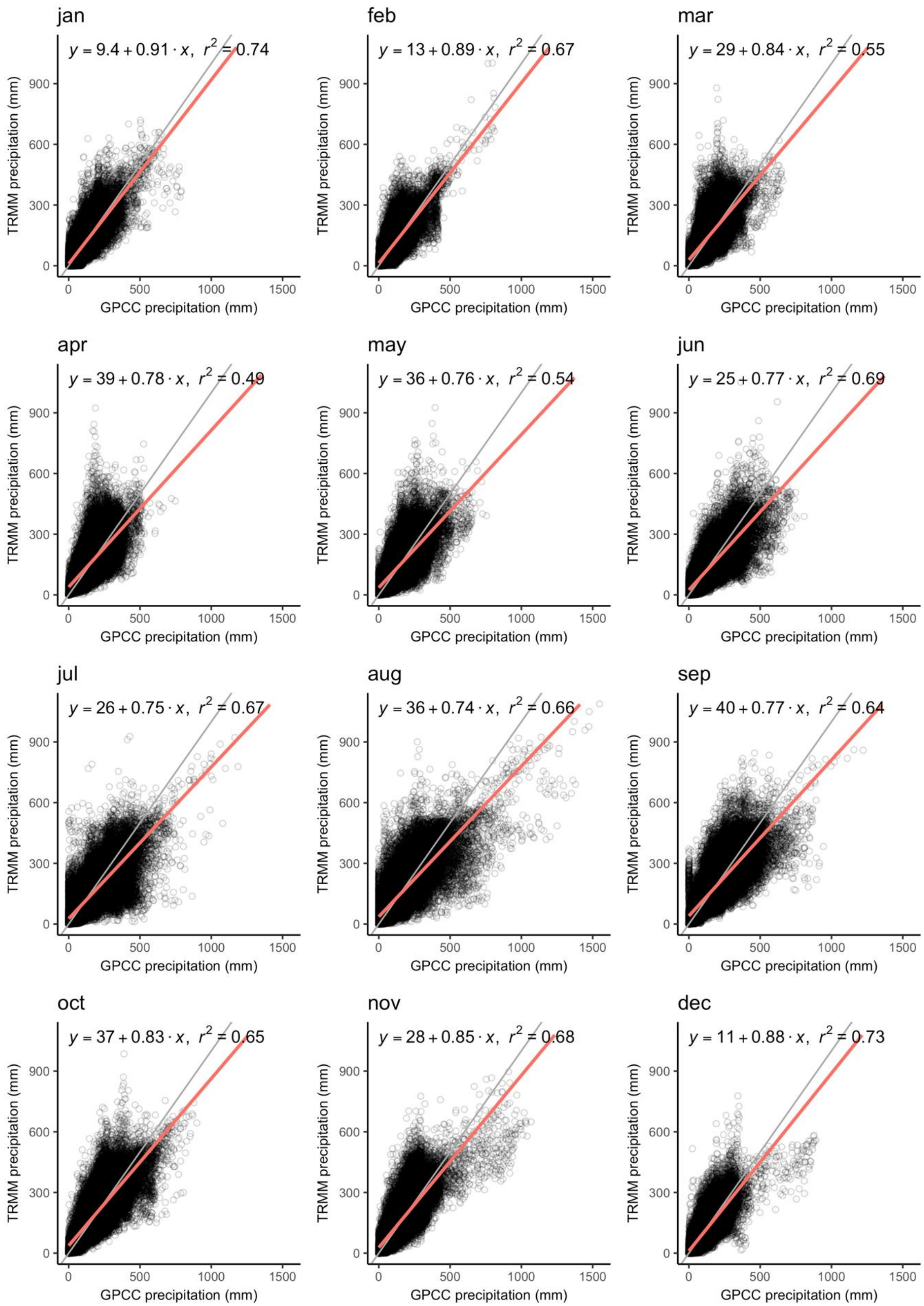

Fig. S2. Monthly correction coefficients for precipitation.

Table S1. Plot attributes.

						Effort			Pre-El Niño interval		El Niño interval	
**Country**	**Cluster**	**Plot Code**	**Lat.**	**Long.**	**Alt. (masl)**	**Area (ha)**	**n censuses**	**Interval (y)**	**Start**	**End**	**Start**	**End**
GHA	ANK	ANK-01	5.27	-2.69	114	1	4	5.6	2011.5	2013.9	2013.9	2017.1
		ANK-02	5.27	-2.7	116	1	4	5.6	2011.5	2013.9	2013.9	2017.1
		ANK-03	5.27	-2.69	86	0.96	4	5.3	2011.8	2013.9	2013.9	2017.1
	ASN	ASN-02	6.56	-2.22	270	0.2	4	15.8	2001.3	2013.9	2013.9	2017.1
	BOB	BOB-01	6.7	-1.32	268	1	4	5.0	2012.2	2015.4	2015.4	2017.2
		BOB-02	6.75	-1.34	276	1	4	5.1	2012.1	2015.4	2015.4	2017.2
		BOB-03	6.69	-1.29	301	1	4	4.9	2012.3	2015.4	2015.4	2017.2
	CAP	CAP-09	4.85	-2.04	126	1	4	16.1	2001.1	2014.0	2014.0	2017.2
		CAP-10	4.8	-2.05	68	1	4	16.1	2001.1	2013.9	2013.9	2017.2
LBR	CVL	CVL-01	6.19	-8.18	257	0.89	3	8.0	2008.8	2014.2	2014.2	2016.9
		CVL-11	6.19	-8.18	260	0.85	3	7.8	2009.1	2014.2	2014.2	2016.9
GHA	DAD	DAD-03	5.99	-3.01	148	1	4	16.0	2001.2	2013.9	2013.9	2017.1
		DAD-04	5.99	-3.02	148	1	4	16.0	2001.2	2013.9	2013.9	2017.1
CMR	DJK	DJK-01	3.33	12.72	647	1	12	12.1	2005.1	2015.2	2015.2	2017.2
		DJK-02	3.33	12.72	722	1	12	12.0	2005.2	2015.2	2015.2	2017.2
		DJK-03	3.36	12.72	639	1	12	12.0	2005.2	2015.2	2015.2	2017.2
		DJK-04	3.36	12.73	639	1	12	12.0	2005.2	2015.2	2015.2	2017.2
		DJK-05	3.32	12.76	779	1	12	12.0	2005.2	2015.2	2015.2	2017.2
		DJK-06	3.33	12.76	639	1	12	12.0	2005.2	2015.2	2015.2	2017.2
	DJL	DJL-01	3.12	13.58	544	1	4	10.6	2006.3	2015.8	2015.8	2016.9
CMR	DJL	DJL-02	3.12	13.59	606	1	4	10.6	2006.3	2015.8	2015.8	2016.9
		DJL-03	3.04	13.62	569	1	4	10.6	2006.3	2015.8	2015.8	2016.9
		DJL-04	3.05	13.62	595	1	4	10.6	2006.3	2015.8	2015.8	2016.9
		DJL-05	3.03	13.58	604	1	4	10.5	2006.4	2015.8	2015.8	2016.9
		DJL-06	3.03	13.61	585	1	4	10.6	2006.4	2015.8	2015.8	2017.0
	DNG	DNG-01	5.21	13.45	NA	1	3	5.3	2011.5	2014.5	2014.5	2016.8
		DNG-02	5.21	13.45	716	1	3	5.2	2011.6	2014.6	2014.6	2016.8
LBR	GBO	GBO-02	5.4	-7.62	172	1	3	8.0	2008.9	2014.3	2014.3	2016.9
		GBO-04	5.4	-7.61	175	0.69	3	6.9	2010.0	2014.3	2014.3	2016.9

		GBO-08	5.39	-7.6	174	1	3	8.0	2008.9	2014.3	2014.3	2016.9
		GBO-11	5.39	-7.59	175	0.67	3	7.8	2009.2	2014.2	2014.2	2016.9
		GBO-15	5.41	-7.61	175	0.71	3	6.9	2010.0	2014.2	2014.2	2016.9
		GBO-19	5.41	-7.6	175	0.78	3	7.8	2009.1	2014.2	2014.2	2016.9
GAB	HAB	HAB-03	0.63	10.97	396	1	6	16.6	2000.3	2014.2	2014.2	2016.9
		HAB-06	0.54	11.14	550	1	5	16.8	2000.4	2014.2	2014.2	2017.2
		HAB-07	0.53	11.17	505	0.8	5	16.7	2000.4	2014.1	2014.1	2017.1
	IVI	IVI-01	0.18	12.54	500	1	4	5.5	2011.5	2015.6	2015.6	2017.0
		IVI-02	0.17	12.53	570	1	4	5.4	2011.5	2015.6	2015.6	2016.9
COG	KOL	KOL-01	1.2	17.88	336	1	3	5.1	2012.1	2014.3	2014.3	2017.2
		KOL-02	1.19	17.84	325	0.6	3	5.1	2012.1	2014.3	2014.3	2017.2
		KOL-03	1.19	17.85	334	0.6	3	5.1	2012.1	2014.3	2014.3	2017.2
		KOL-04	1.23	17.91	329	1	3	5.1	2012.1	2014.3	2014.3	2017.2
COD	KSN	KSN-01	0.3	25.31	449	0.2	3	8.6	2008.6	2013.6	2013.6	2017.2
		KSN-02	0.31	25.31	455	0.2	3	8.6	2008.6	2013.6	2013.6	2017.2
COD	KSN	KSN-05	0.31	25.31	452	0.2	3	8.6	2008.6	2013.6	2013.6	2017.2
		KSN-06	0.31	25.31	440	0.2	3	8.6	2008.6	2013.7	2013.7	2017.2
COG	LTL	LTL-01	1.36	17.43	339	1	3	7.0	2010.1	2014.4	2014.4	2017.1
GAB	MDC	MDC-01	0.62	10.41	647	1	3	12.5	2004.4	2013.6	2013.6	2016.9
		MDC-02	0.62	10.41	623	1	4	12.5	2004.4	2013.6	2013.6	2016.9
		MDC-03	0.61	10.4	494	1	4	12.5	2004.4	2014.1	2014.1	2016.9
		MDC-04	0.47	10.28	136	1	4	12.4	2004.4	2013.2	2013.2	2016.8
		MDC-05	0.46	10.28	210	1	3	12.7	2004.4	2013.2	2013.2	2017.1
CMR	MDJ	MDJ-01	6.17	12.83	789	1	3	9.2	2007.8	2014.1	2014.1	2017.0
		MDJ-03	5.98	12.87	757	1	3	9.2	2007.8	2014.1	2014.1	2017.0
		MDJ-07	6.01	12.89	764	1	3	9.1	2007.9	2014.2	2014.2	2017.0
		MDJ-10	6	12.89	767	1	3	9.1	2007.9	2014.2	2014.2	2017.0
GAB	MNG	MNG-03	0.57	9.33	64	1	5	5.3	2011.8	2015.5	2015.5	2017.1
		MNG-04	0.58	9.32	57	1	5	4.8	2012.0	2015.5	2015.5	2016.8
COG	NNN	NNN-01	2.66	16.62	460	1	7	7.1	2010.1	2015.1	2015.1	2017.2
		NNN-02	2.61	16.53	509	1	7	7.0	2010.2	2015.1	2015.1	2017.2
		NNN-03	2.64	16.55	476	1	7	7.0	2010.2	2015.1	2015.1	2017.2

		NNN-04	2.4	16.47	459	1	7	7.2	2009.9	2015	2015.0	2017.1
		NNN-05	2.36	16.43	429	1	7	7.2	2009.9	2014.9	2014.9	2017.1
		NNN-06	2.36	16.36	444	1	7	7.2	2009.9	2015	2015	2017.1
	NNP	NNP-01	2.23	16.4	394	1	3	11.6	2005.5	2014.3	2014.3	2017.1
		NNP-02	2.24	16.4	404	1	3	11.6	2005.5	2014.3	2014.3	2017.1
		NNP-05	2.2	16.36	410	1	3	11.6	2005.5	2014.3	2014.3	2017.1
GAB	OVG	OVG-01	0.73	11.37	580	1	3	9.9	2006.9	2013.5	2013.5	2016.8
COG	SAN	SAN-22	2.3	16.39	416	1	4	11.7	2005.6	2013.5	2013.5	2017.3
		SAN-24	2.34	16.35	397	1	4	11.7	2005.6	2013.6	2013.6	2017.3
COD	SNG	SNG-01	-1.7	20.55	371	1	3	8.9	2008.3	2014.5	2014.5	2017.2
		SNG-02	-1.7	20.55	365	1	3	9.0	2008.3	2014.6	2014.6	2017.3
		SNG-03	-1.7	20.55	420	1	3	8.9	2008.4	2014.6	2014.6	2017.3
		SNG-04	-1.7	20.54	384	1	3	8.9	2008.4	2014.6	2014.6	2017.3
		SNG-05	-1.7	20.54	361	1	3	8.9	2008.4	2014.6	2014.6	2017.3
		SNG-06	-1.7	20.54	360	1	3	8.9	2008.4	2014.6	2014.6	2017.3
		SNG-07	-1.7	20.54	362	1	3	8.9	2008.4	2014.6	2014.6	2017.3
		SNG-08	-1.7	20.54	382	1	3	8.9	2008.4	2014.6	2014.6	2017.3
		SNG-09	-1.7	20.54	374	1	3	8.9	2008.4	2014.6	2014.6	2017.3
	YGB	YGB-08	0.8	24.49	460	0.8	3	4.9	2012.3	2014.3	2014.3	2017.2
		YGB-14	0.83	24.52	438	1.07	3	4.8	2012.3	2014.3	2014.3	2017.1
		YGB-15	0.83	24.53	464	1.07	3	4.8	2012.3	2014.3	2014.3	2017.1
		YGB-16	0.8	24.5	427	0.75	3	4.9	2012.3	2014.3	2014.3	2017.2
		YGB-17	0.81	24.53	466	0.92	3	4.9	2012.3	2014.3	2014.3	2017.2
		YGB-18	0.87	24.46	427	1.01	3	4.9	2012.3	2014.3	2014.3	2017.2
		YGB-24	0.81	24.51	464	1.07	3	4.8	2012.3	2014.3	2014.3	2017.1
		YGB-25	0.78	24.52	477	0.8	3	4.9	2012.3	2014.3	2014.3	2017.2
		YGB-26	0.79	24.52	435	0.69	3	4.9	2012.3	2014.3	2014.3	2017.2
		YGB-27	0.81	24.49	417	0.92	3	4.8	2012.3	2014.3	2014.3	2017.1
		YGB-28	0.8	24.49	489	0.72	3	4.9	2012.3	2014.3	2014.3	2017.2
	YOK	YOK-06	0.29	25.33	439	1	3	7.6	2009.5	2013.6	2013.6	2017.1
		YOK-07	0.29	25.33	433	1	3	7.6	2009.5	2013.6	2013.6	2017.1
COD	YOK	YOK-08	0.29	25.33	433	1	3	7.6	2009.5	2013.6	2013.6	2017.1

		YOK-09	0.3	25.33	431	1	3	7.6	2009.5	2013.6	2013.6	2017.1
		YOK-10	0.3	25.33	418	1	3	7.6	2009.5	2013.6	2013.6	2017.1
		YOK-16	0.29	25.33	450	0.88	3	7.6	2009.5	2013.7	2013.7	2017.1
		YOK-17	0.29	25.33	440	0.75	3	7.6	2009.5	2013.7	2013.7	2017.1
		YOK-18	0.3	25.33	441	0.75	3	7.6	2009.5	2013.7	2013.7	2017.1
		YOK-19	0.3	25.33	441	0.75	3	7.6	2009.5	2013.7	2013.7	2017.1
		YOK-20	0.3	25.33	435	0.88	3	7.7	2009.5	2013.7	2013.7	2017.2

Table S2. Parameters used to estimate tree height from tree diameter.

PlotCode	a	b	c
ANK-01	54.9	0.05	0.69
ANK-02	54.9	0.05	0.69
ANK-03	54.9	0.05	0.69
ASN-02	54.9	0.05	0.69
BOB-01	54.9	0.05	0.69
BOB-02	54.9	0.05	0.69
BOB-03	54.9	0.05	0.69
CAP-09	54.9	0.05	0.69
CAP-10	54.9	0.05	0.69
CVL-01	54.9	0.05	0.69
CVL-11	54.9	0.05	0.69
DAD-03	54.9	0.05	0.69
DAD-04	54.9	0.05	0.69
DJK-01	44.0	0.05	0.85
DJK-02	44.0	0.05	0.85
DJK-03	44.0	0.05	0.85
DJK-04	44.0	0.05	0.85
DJK-05	44.0	0.05	0.85
DJK-06	44.0	0.05	0.85
DJL-01	44.0	0.05	0.85
DJL-02	44.0	0.05	0.85
DJL-03	44.0	0.05	0.85
DJL-04	44.0	0.05	0.85
DJL-05	44.0	0.05	0.85
DJL-06	44.0	0.05	0.85
DNG-01	44.0	0.05	0.85
DNG-02	44.0	0.05	0.85
GBO-02	54.9	0.05	0.69
GBO-04	54.9	0.05	0.69
GBO-08	54.9	0.05	0.69
GBO-11	54.9	0.05	0.69
GBO-15	54.9	0.05	0.69
GBO-19	54.9	0.05	0.69
HAB-03	44.0	0.05	0.85
HAB-06	44.0	0.05	0.85
HAB-07	44.0	0.05	0.85
IVI-01	44.0	0.05	0.85

IVI-02	44.0	0.05	0.85
KOL-01	44.0	0.05	0.85
KOL-02	79.2	0.04	0.55
KOL-03	79.2	0.04	0.55
KOL-04	44.0	0.05	0.85
KSN-01	48.2	0.05	0.71
KSN-02	48.2	0.05	0.71
KSN-05	48.2	0.05	0.71
KSN-06	48.2	0.05	0.71
LTL-01	79.2	0.04	0.55
MDC-01	44.0	0.05	0.85
MDC-02	44.0	0.05	0.85
MDC-03	44.0	0.05	0.85
MDC-04	44.0	0.05	0.85
MDC-05	44.0	0.05	0.85
MDJ-01	44.0	0.05	0.85
MDJ-03	44.0	0.05	0.85
MDJ-07	44.0	0.05	0.85
MDJ-10	44.0	0.05	0.85
MNG-03	44.0	0.05	0.85
MNG-04	44.0	0.05	0.85
NNN-01	44.0	0.05	0.85
NNN-02	44.0	0.05	0.85
NNN-03	44.0	0.05	0.85
NNN-04	44.0	0.05	0.85
NNN-05	44.0	0.05	0.85
NNN-06	44.0	0.05	0.85
NNP-01	44.0	0.05	0.85
NNP-02	44.0	0.05	0.85
NNP-05	44.0	0.05	0.85
OVG-01	44.0	0.05	0.85
SAN-22	44.0	0.05	0.85
SAN-24	44.0	0.05	0.85
SNG-01	48.2	0.05	0.71
SNG-02	48.2	0.05	0.71
SNG-03	48.2	0.05	0.71
SNG-04	48.2	0.05	0.71
SNG-05	48.2	0.05	0.71
SNG-06	48.2	0.05	0.71

SNG-07	48.2	0.05	0.71
SNG-08	48.2	0.05	0.71
SNG-09	48.2	0.05	0.71
YGB-08	48.2	0.05	0.71
YGB-14	48.2	0.05	0.71
YGB-15	48.2	0.05	0.71
YGB-16	48.2	0.05	0.71
YGB-17	48.2	0.05	0.71
YGB-18	48.2	0.05	0.71
YGB-24	48.2	0.05	0.71
YGB-25	48.2	0.05	0.71
YGB-26	48.2	0.05	0.71
YGB-27	48.2	0.05	0.71
YGB-28	48.2	0.05	0.71
YOK-06	48.2	0.05	0.71
YOK-07	48.2	0.05	0.71
YOK-08	48.2	0.05	0.71
YOK-09	48.2	0.05	0.71
YOK-10	48.2	0.05	0.71
YOK-16	48.2	0.05	0.71
YOK-17	48.2	0.05	0.71
YOK-18	48.2	0.05	0.71
YOK-19	48.2	0.05	0.71
YOK-20	48.2	0.05	0.71

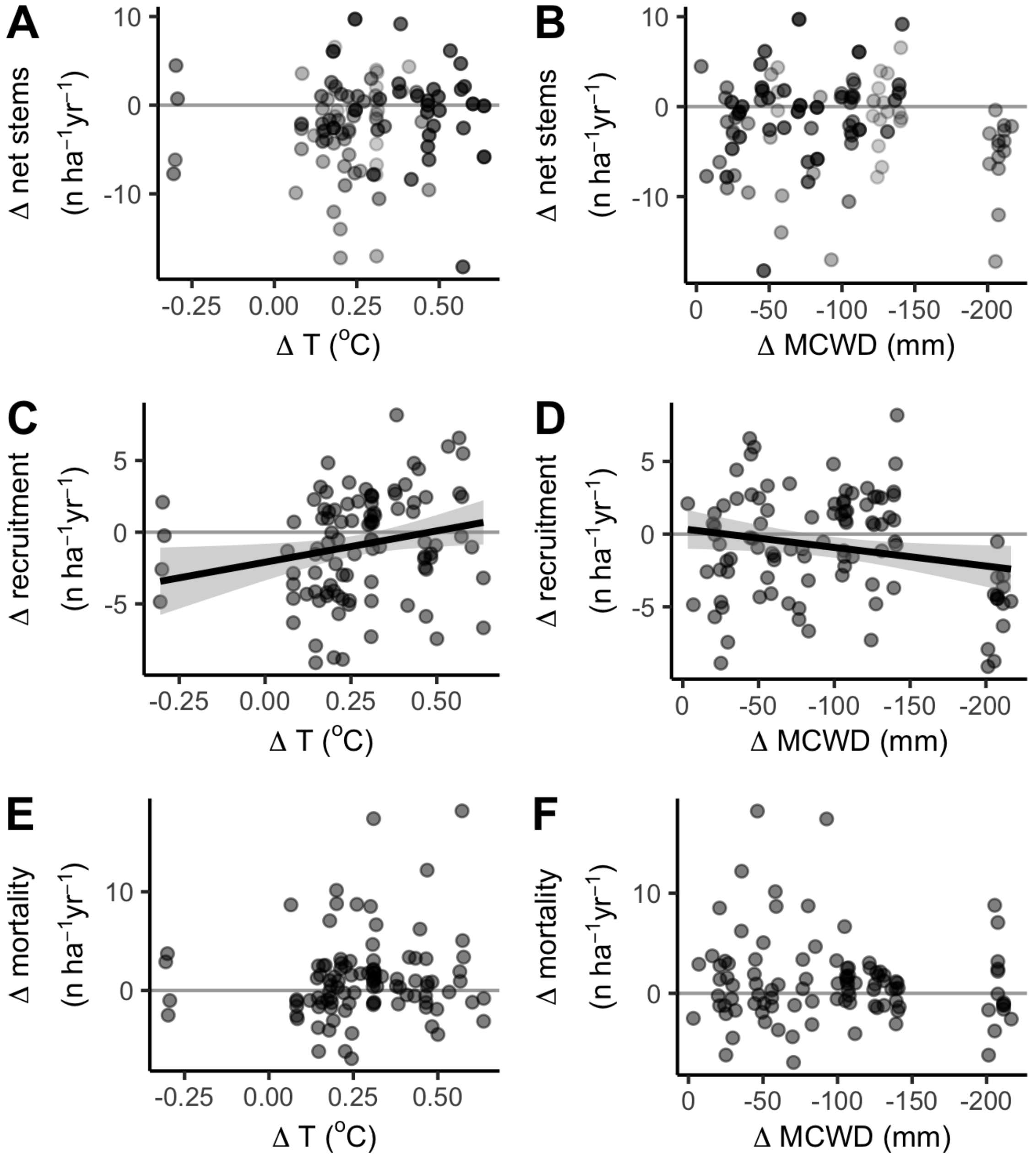

Fig. S3. Temperature (left) and drought (right) impacts on stem dynamics

The net stems change (A, B), stem gains from newly recruited stems (C, D) and stem losses from tree mortality (E, F) of the censuses capturing the El Niño event minus the pre-El Niño plot monitoring period for 100 long-term inventory plots. The relative intensity of temperature change, Δ temperature (T) (A, C, E) is mean monthly temperature in El Niño minus mean monthly temperature pre-El Niño, using the census dates of the plot censuses. Relative intensity of the change in dry season strength, is calculated as Δ maximum cumulative water deficit (MCWD) (B, D, F) which is the difference between maximum MCWD in El Niño and mean MCWD in pre-El Niño. Point shading from light to dark denotes greater weighting, with plots and line of best fit weighted by an empirically derived combination of pre-El Niño plot monitoring length and plot area for each response variable. Solid lines represent significant linear models ($p \leq 0.05$) with 95 % confidence intervals (shading around line).

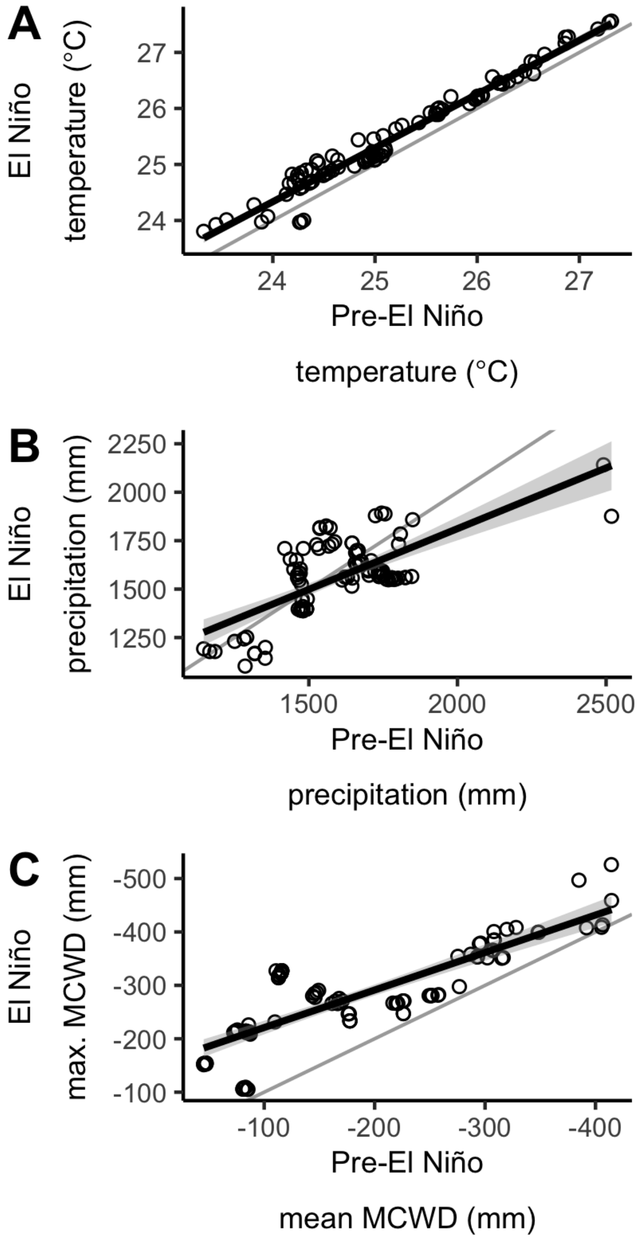

Fig. S4. Climate anomalies of 100 long-term inventory plots. Pre- El Niño and El Niño are defined by plot census dates.

Plot census interval pre-El Niño and El Niño temperature (A), linear model $p < 0.0001$, plot census interval monthly precipitation (B), $p < 0.0001$, and plot census interval maximum cumulative water deficit MCWD (C), $p < 0.0001$ in plots with 95 % confidence interval (shading around line). Grey line indicates 1:1 relationship. n=100 plots censused pre- and El Niño.

Table S3. Coefficients of model-averaged multiple regression models of net carbon, carbon gains and carbon losses.

	Δ net carbon				Δ carbon gains				Δ carbon losses			
Variable	Estimate	SE	Z	P	Estimate	SE	Z	P	Estimate	SE	Z	P
Intercept	0.14	0.69	0.2	0.8	-0.20	0.14	1.5	0.1	0.14	0.69	0.2	0.8
Pre-El Niño temperature	0.05	0.42	0.1	0.9	0.01	0.07	0.2	0.9	0.05	0.42	0.1	0.9
Pre-El Niño MCWD	0.02	0.53	0.03	0.9	0.01	0.08	0.1	0.9	0.02	0.53	0.03	0.9
Δ temperature	0.91	0.99	0.9	0.4	0.04	0.11	0.3	0.7	0.91	0.99	0.9	0.4
Δ MCWD	1.19	0.78	0.9	0.4	-0.09	0.14	0.7	0.5	1.20	0.78	1.5	0.1
Interaction: Δ temperature and Δ MCWD	0.55	0.81	0.7	0.5	0.01	0.06	0.2	0.9	0.55	0.81	0.7	0.5
Interaction: Δ temperature and pre-El Niño temperature	0.05	0.33	0.2	0.9	0.006	0.05	0.1	0.9	0.05	0.33	0.2	0.9
Interaction: Δ MCWD and pre-El Niño MCWD	-0.57	0.97	0.6	0.6	0.003	0.04	0.1	0.9	-0.57	0.97	0.6	0.6

Table S4. Coefficients of model-averaged multiple regression models of net stems, recruitment and stem mortality.

	Δ net stems				Δ recruitment				Δ mortality			
Variable	Estimate	SE	Z	P	Estimate	SE	Z	P	Estimate	SE	Z	P
Intercept	-0.81	0.54	3.3	<0.001	-0.70	0.39	1.8	0.07	1.09	0.48	2.2	≤0.05
Pre-El Niño temperature	-0.001	0.26	0.005	0.9	0.10	0.26	0.4	0.7	0.25	0.43	0.6	0.6
Pre-El Niño MCWD	-0.12	0.38	0.3	0.8	-0.21	0.38	0.6	0.6	0.53	0.53	1.0	0.3
Δ temperature	0.12	0.39	0.3	0.8	1.37	0.68	2.0	0.05	0.27	0.47	0.6	0.6
Δ MCWD	-0.04	0.35	0.1	0.9	-0.22	0.53	0.4	0.7	-0.06	0.32	0.2	0.9
Interaction: Δ temperature and Δ MCWD	0.03	0.22	0.1	0.9	0.84	0.68	1.2	0.2	0.003	0.07	0.04	0.9
Interaction: Δ temperature and pre-El Niño temperature	-0.002	0.09	0.02	0.9	0.07	0.29	0.2	0.8	0.02	0.18	0.1	0.9
Interaction: Δ MCWD and pre-El Niño MCWD	0.08	0.36	0.2	0.8	0.02	0.23	0.1	0.9	-0.12	0.49	0.2	0.8

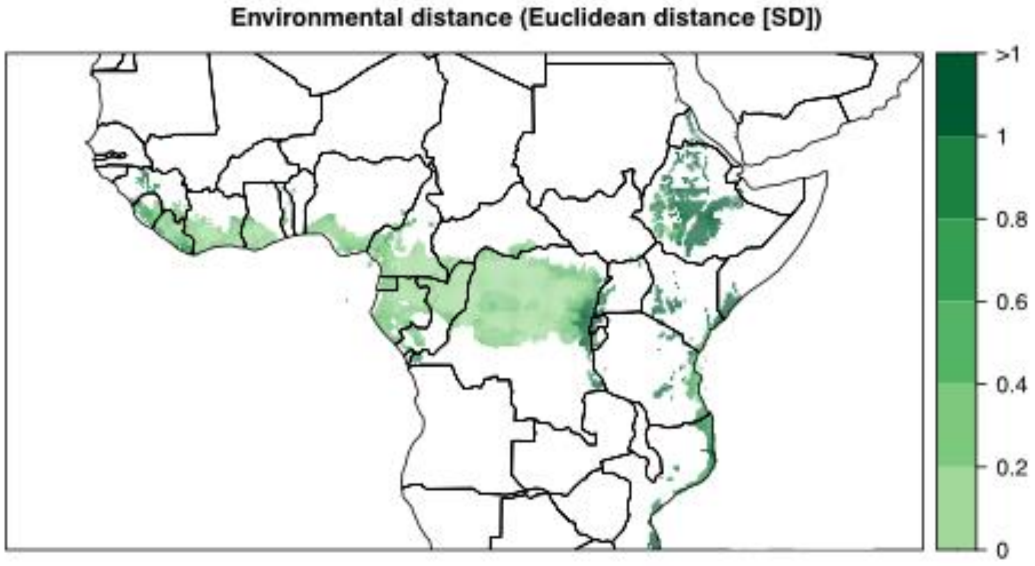

Fig. S5. Representativeness of 100 plots.

The minimum environmental distance (Euclidean distance, in units of standard deviation) between each location in the tropical forest biome and the 100 plots. Environmental variables used are mean annual temperature, temperature seasonality, total annual precipitation and precipitation seasonality.

Table S5. Aboveground biomass carbon calculated using the BIOMASS R package.

Biomass is calculated using the AGBmonteCarlo function and then converted to carbon using the mean carbon fraction of aboveground biomass for tropical angiosperms, 45.6 % (1).

**PlotCode**	**Pre-El Niño biomass carbon** Mg C ha^{-1} ± 95 % CI	**El Niño biomass carbon** Mg C ha^{-1} ± 95 % CI
ANK-01	166 ± 35	177 ± 35
ANK-02	166 ± 33	168 ± 32
ANK-03	134 ± 33	144 ± 33
ASN-02	143 ± 38	147 ± 37
BOB-01	108 ± 30	111 ± 29
BOB-02	117 ± 38	127 ± 39
BOB-03	108 ± 35	113 ± 35
CAP-09	182 ± 40	184 ± 39
CAP-10	107 ± 28	127 ± 30
CVL-01	153 ± 33	154 ± 32
CVL-11	164 ± 34	165 ± 33
DAD-03	58 ± 17	45 ± 14
DAD-04	149 ± 32	138 ± 30
DJK-01	200 ± 37	215 ± 37
DJK-02	143 ± 32	153 ± 32
DJK-03	200 ± 38	212 ± 38
DJK-04	112 ± 29	119 ± 29
DJK-05	211 ± 40	228 ± 41
DJK-06	117 ± 28	127 ± 30
DJL-01	234 ± 44	253 ± 45
DJL-02	173 ± 37	188 ± 38
DJL-03	270 ± 51	295 ± 53
DJL-04	159 ± 37	170 ± 38
DJL-05	228 ± 42	247 ± 43
DJL-06	126 ± 32	129 ± 31
DNG-01	210 ± 55	231 ± 57
DNG-02	170 ± 45	184 ± 46
GBO-02	171 ± 34	184 ± 35
GBO-04	155 ± 34	159 ± 34
GBO-08	162 ± 33	162 ± 32
GBO-11	135 ± 29	146 ± 30

GBO-15	143 ± 31	154 ± 32
GBO-19	157 ± 35	169 ± 36
HAB-03	184 ± 39	202 ± 40
HAB-06	149 ± 37	181 ± 40
HAB-07	254 ± 58	265 ± 57
IVI-01	183 ± 36	189 ± 35
IVI-02	177 ± 34	187 ± 34
KOL-01	149 ± 30	154 ± 29
KOL-02	71 ± 17	81 ± 18
KOL-03	141 ± 34	140 ± 32
KOL-04	233 ± 42	251 ± 43
KSN-01	208 ± 42	227 ± 44
KSN-02	129 ± 30	144 ± 33
KSN-05	170 ± 35	181 ± 35
KSN-06	169 ± 36	179 ± 36
LTL-01	165 ± 38	173 ± 38
MDC-01	144 ± 36	158 ± 37
MDC-02	195 ± 45	178 ± 38
MDC-03	216 ± 42	222 ± 42
MDC-04	212 ± 41	218 ± 41
MDC-05	216 ± 43	232 ± 43
MDJ-01	168 ± 43	182 ± 44
MDJ-03	119 ± 31	127 ± 31
MDJ-07	120 ± 31	134 ± 32
MDJ-10	69 ± 21	82 ± 23
MNG-03	218 ± 57	230 ± 57
MNG-04	187 ± 42	199 ± 43
NNN-01	213 ± 39	227 ± 38
NNN-02	188 ± 36	195 ± 35
NNN-03	142 ± 33	148 ± 32
NNN-04	190 ± 39	200 ± 39
NNN-05	220 ± 45	239 ± 46
NNN-06	121 ± 27	122 ± 26
NNP-01	170 ± 33	162 ± 31
NNP-02	151 ± 33	160 ± 33
NNP-05	135 ± 30	131 ± 27
OVG-01	214 ± 44	217 ± 42
SAN-22	220 ± 41	224 ± 39
SAN-24	158 ± 35	158 ± 33

SNG-01	161 ± 33	169 ± 33
SNG-02	122 ± 29	137 ± 31
SNG-03	155 ± 32	166 ± 33
SNG-04	134 ± 30	124 ± 29
SNG-05	140 ± 32	151 ± 33
SNG-06	150 ± 33	168 ± 35
SNG-07	99 ± 25	109 ± 26
SNG-08	153 ± 33	165 ± 34
SNG-09	118 ± 29	126 ± 30
YGB-08	199 ± 41	212 ± 41
YGB-14	184 ± 35	191 ± 35
YGB-15	170 ± 36	176 ± 35
YGB-16	187 ± 37	192 ± 37
YGB-17	164 ± 34	170 ± 34
YGB-18	163 ± 31	173 ± 31
YGB-24	179 ± 40	190 ± 41
YGB-25	193 ± 41	202 ± 41
YGB-26	187 ± 38	190 ± 37
YGB-27	213 ± 43	224 ± 43
YGB-28	153 ± 33	167 ± 34
YOK-06	125 ± 31	136 ± 33
YOK-07	150 ± 36	152 ± 35
YOK-08	181 ± 41	413 ± 42
YOK-09	181 ± 41	424 ± 42
YOK-10	180 ± 42	194 ± 44
YOK-16	186 ± 40	145 ± 34
YOK-17	164 ± 36	171 ± 36
YOK-18	132 ± 35	143 ± 36
YOK-19	146 ± 35	159 ± 36
YOK-20	156 ± 35	173 ± 36

Summary of sensitivity analyses

We reproduced Figure 2 and 3 from the main analysis using several alternative methods, Figures S6-S12 are summarised here in Table S6.

Table S6. Summary of sensitivity analyses.

Sensitivity Analyses	Δ net carbon ~ Δ T		Δ carbon gains ~ Δ T		Δ carbon losses ~ Δ T		Δ net carbon ~ Δ MCWD		Δ carbon gains ~ Δ MCWD		Δ carbon losses ~ Δ MCWD	
	Slope	Sig. of linear model	Slope	Sig. of linear model	Slope	Sig. of linear model	Slope	Sig. of linear model	Slope	Sig. of linear model	Slope	Sig. of linear model
Main analysis	-0.29	0.8	0.19	0.6	0.53	0.7	-0.009	**≤0.05**	-0.002	0.10	0.007	0.08
Without weighting by area and time	-0.42	0.8	0.15	0.7	0.66	0.6	-0.009	**≤0.05**	-0.002	0.11	0.007	0.08
Extending pre-El Niño monitoring period to 1984	0.21	0.9	0.19	0.5	0.06	0.9	-0.008	**≤0.05**	-0.002	0.11	0.006	0.11
Talbot census interval correction	-0.30	0.8	0.31	0.3	0.54	0.6	-0.009	**≤0.05**	-0.002	**≤0.05**	0.007	0.09
Δ MCWD calculated with variable ET							-0.009	**≤0.05**	-0.002	**≤0.05**	0.007	0.06
Δ MCWD = El Niño mean MCWD – pre-El Niño mean MCWD							-0.02	**≤0.05**	-0.003	0.16	0.01	0.11
Δ MCWD = El Niño maximum MCWD – pre-El Niño maximum MCWD							-0.006	0.06	-0.002	**≤0.05**	0.004	0.2
Using BIOMASS r package	-0.41	0.8					-0.01	**≤0.05**				
Basal area instead of biomass carbon*	-0.07	0.8	0.04	0.5	0.1	0.6	-0.001	0.08	-0.0004	**≤0.05**	0.0007	0.3
Excluding plots that did not warm	0.55	0.7	0.84	**0.05**	0.41	0.8						

*slopes in different units, m2 ha-1 yr-1, rather than Mg C ha-1 yr-1 in all other cases.

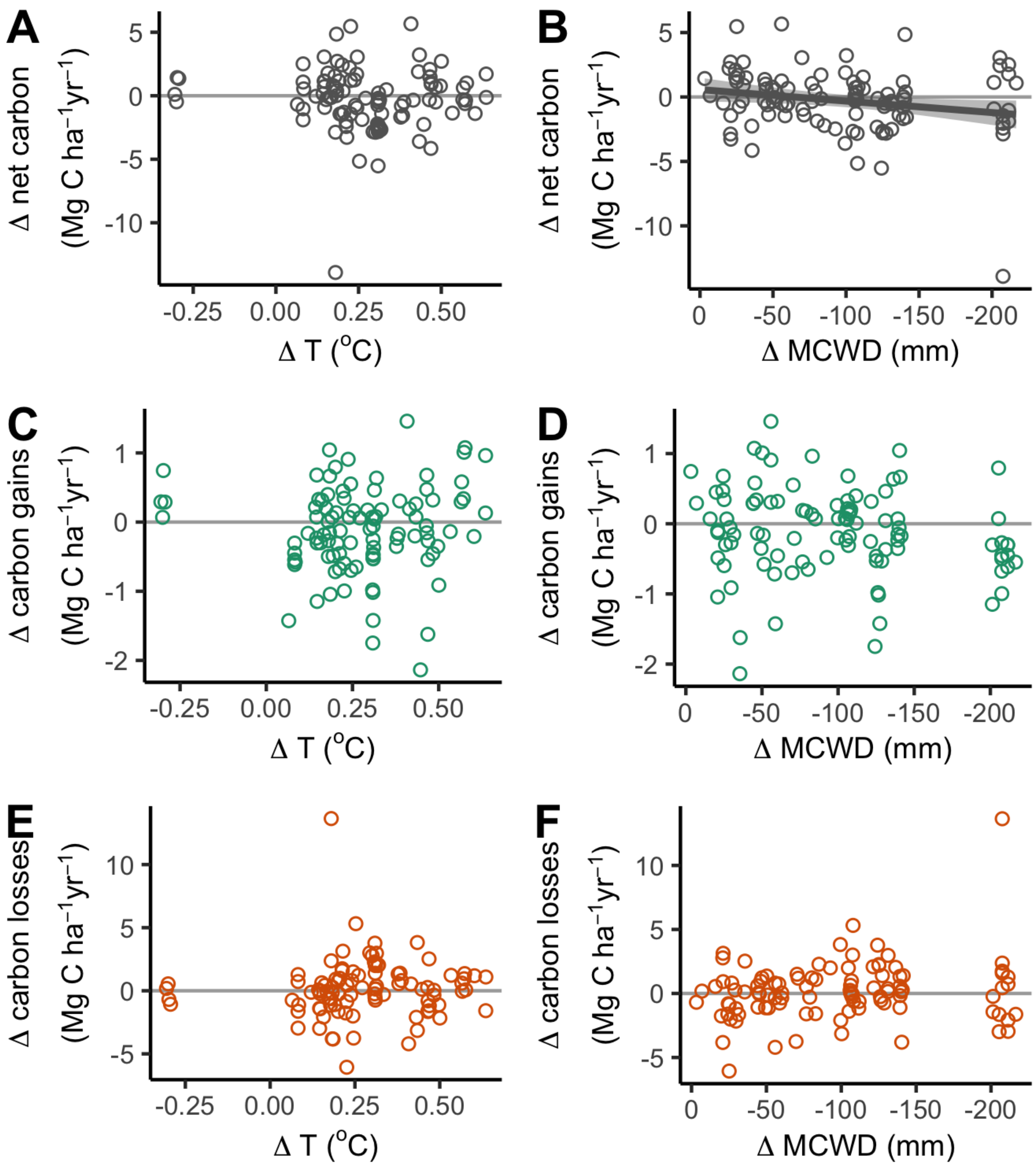

Fig. S6. Figure 2 reproduced without weighting plots by area and pre-El Niño census length.

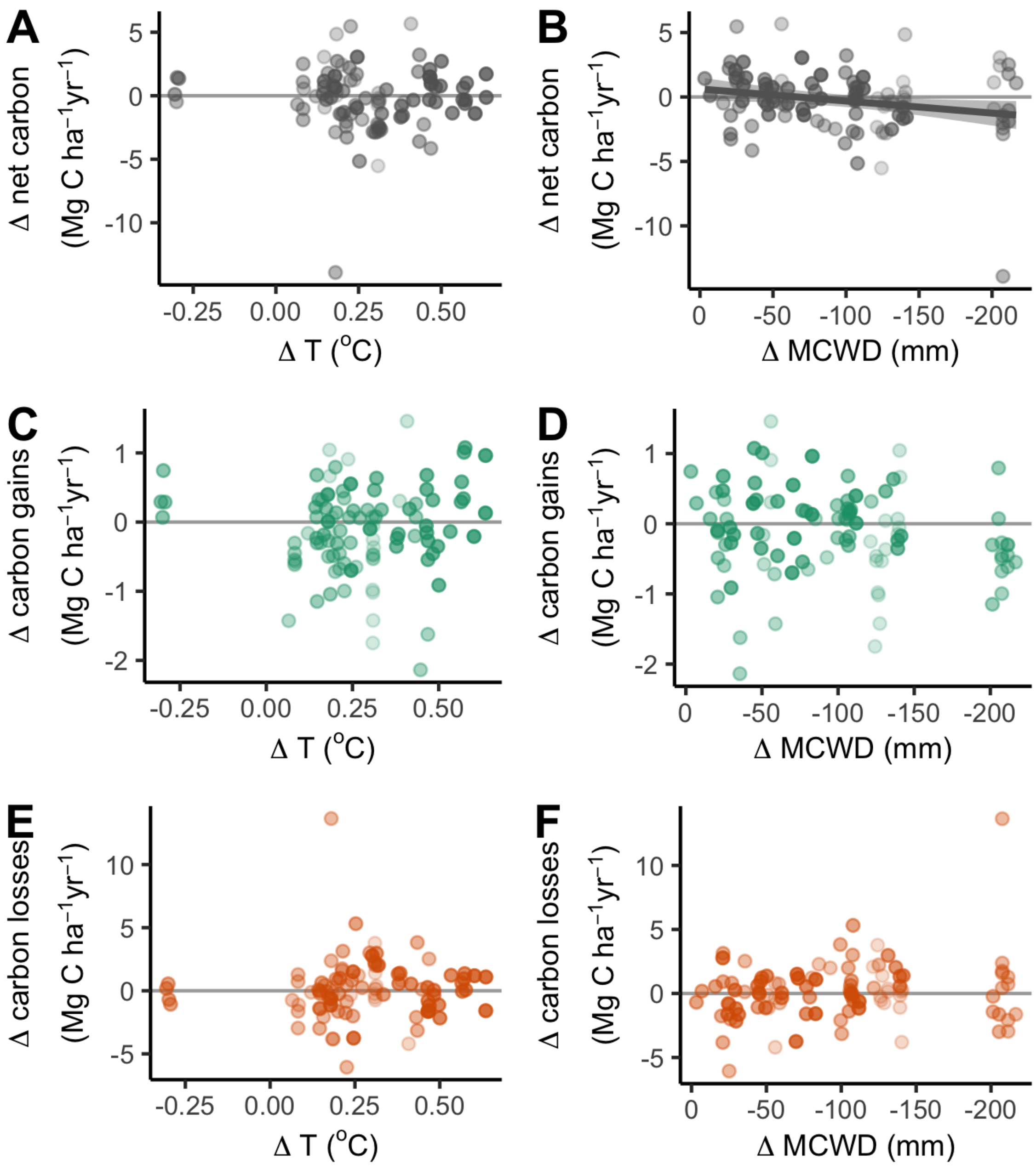

Fig. S7. Figure 2 reproduced using pre-El Niño census intervals back to 1984.

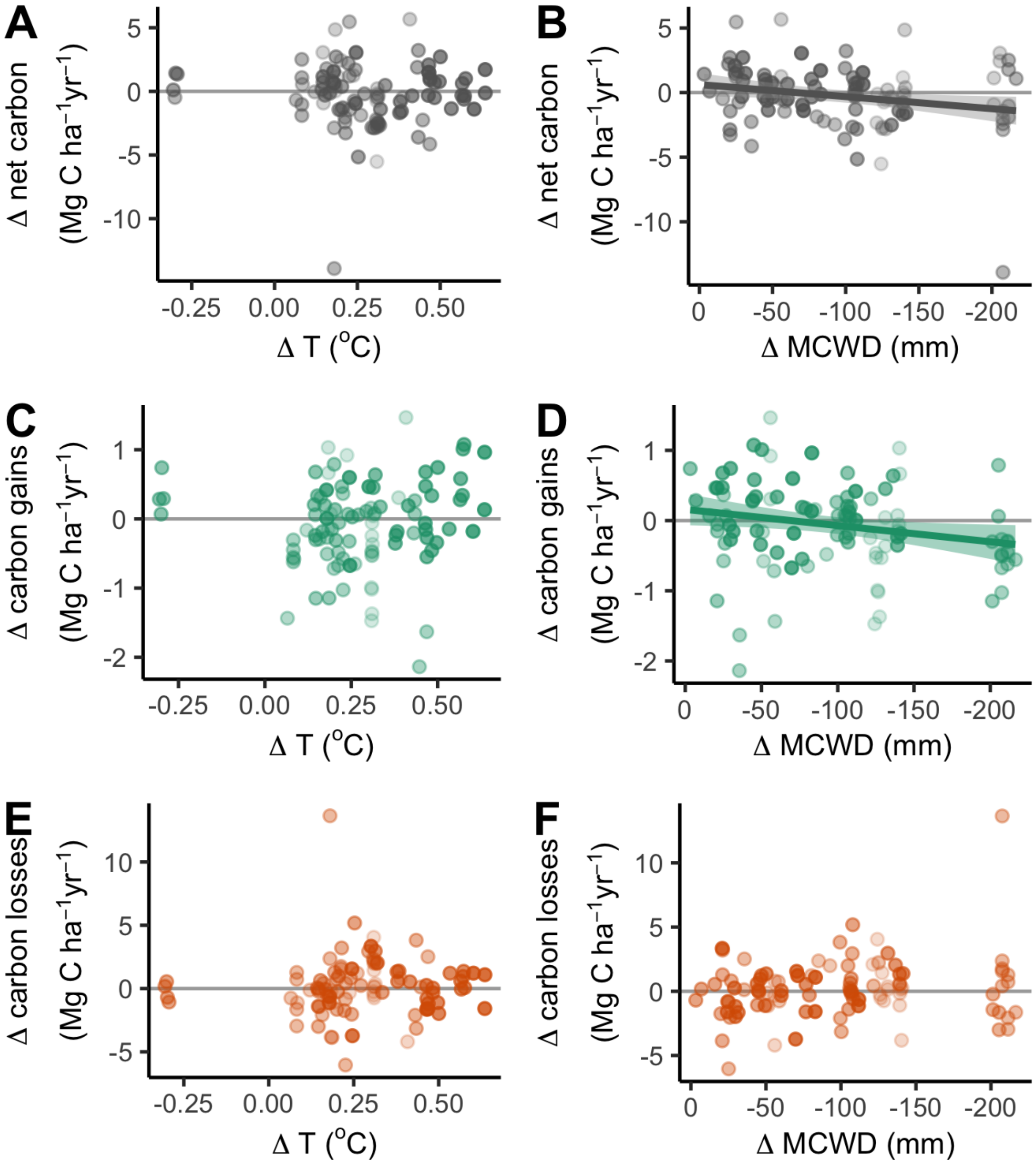

Fig. S8. Figure 2 reproduced with Talbot et al. 2014 (2) census interval correction instead of Kohyama et al. 2019 (3).

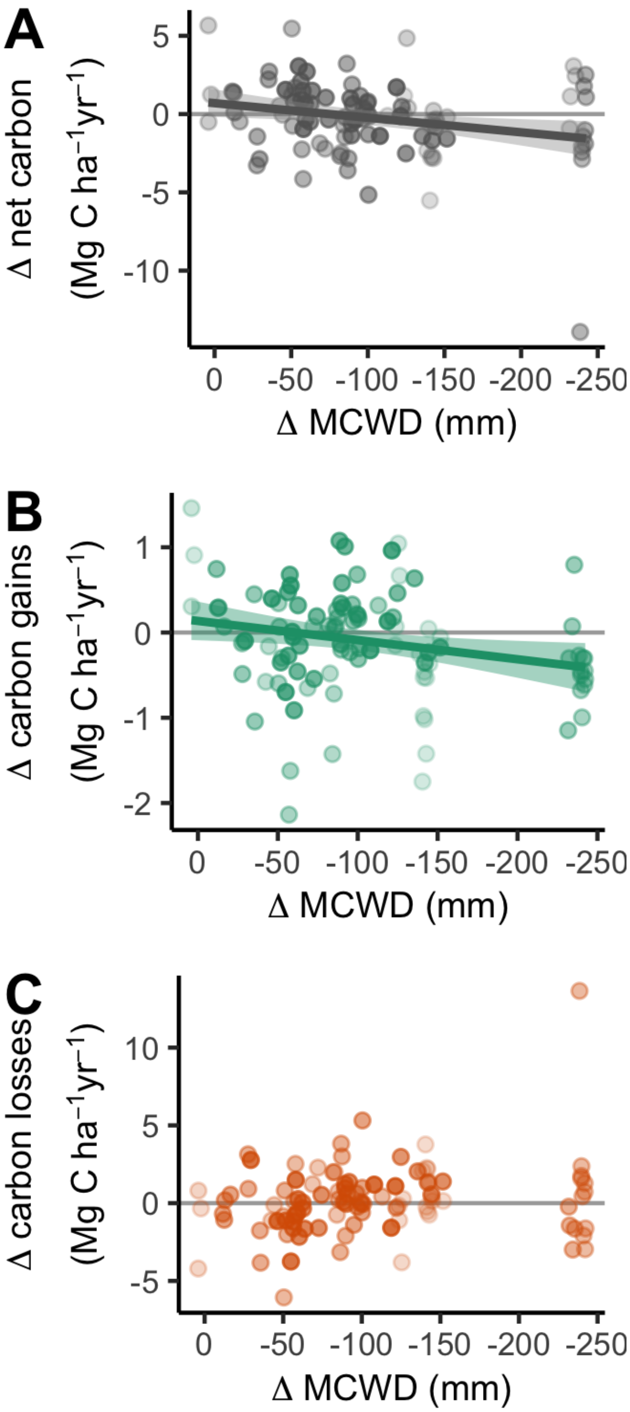

Fig. S9. Figure 2 reproduced with MCWD calculated using variable evapotranspiration derived from rainfall and temperature.

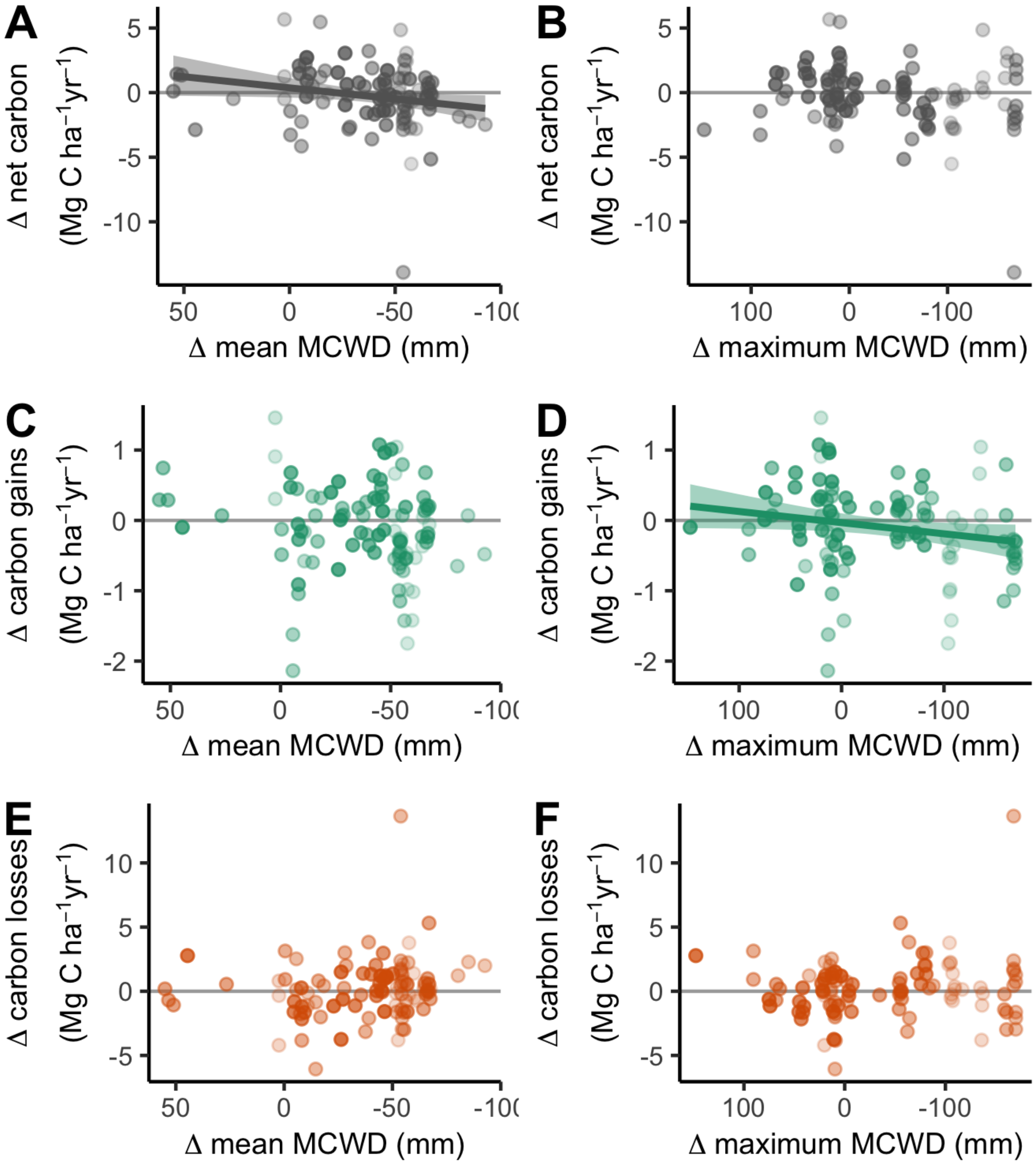

Fig. S10. Figure 2 reproduced with alternative estimates of Δ MCWD.

Δ mean MCWD = El Niño mean MCWD – pre-El Niño mean MCWD (A, C, E) and Δ maximum MCWD = El Niño maximum MCWD – pre-El Niño maximum MCWD (B, D, F).

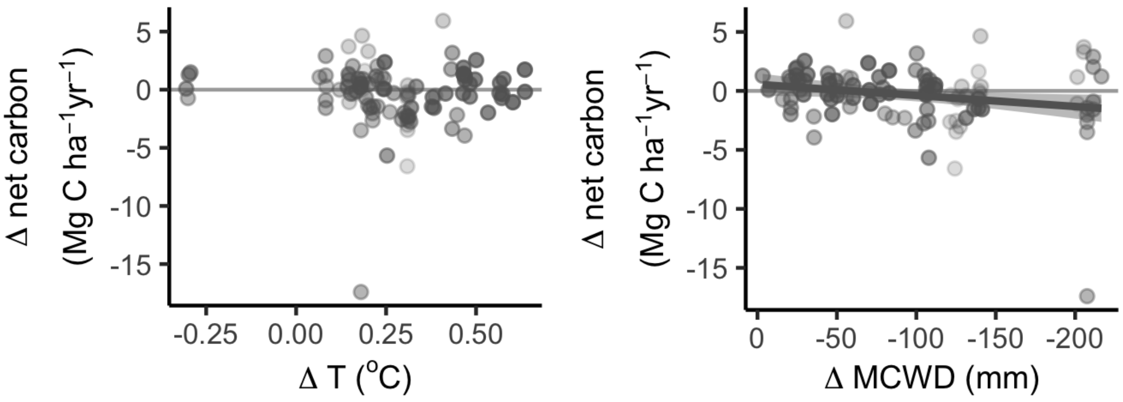

Fig. S11. Figure 2 reproduced with net carbon estimated using the BIOMASS r package.

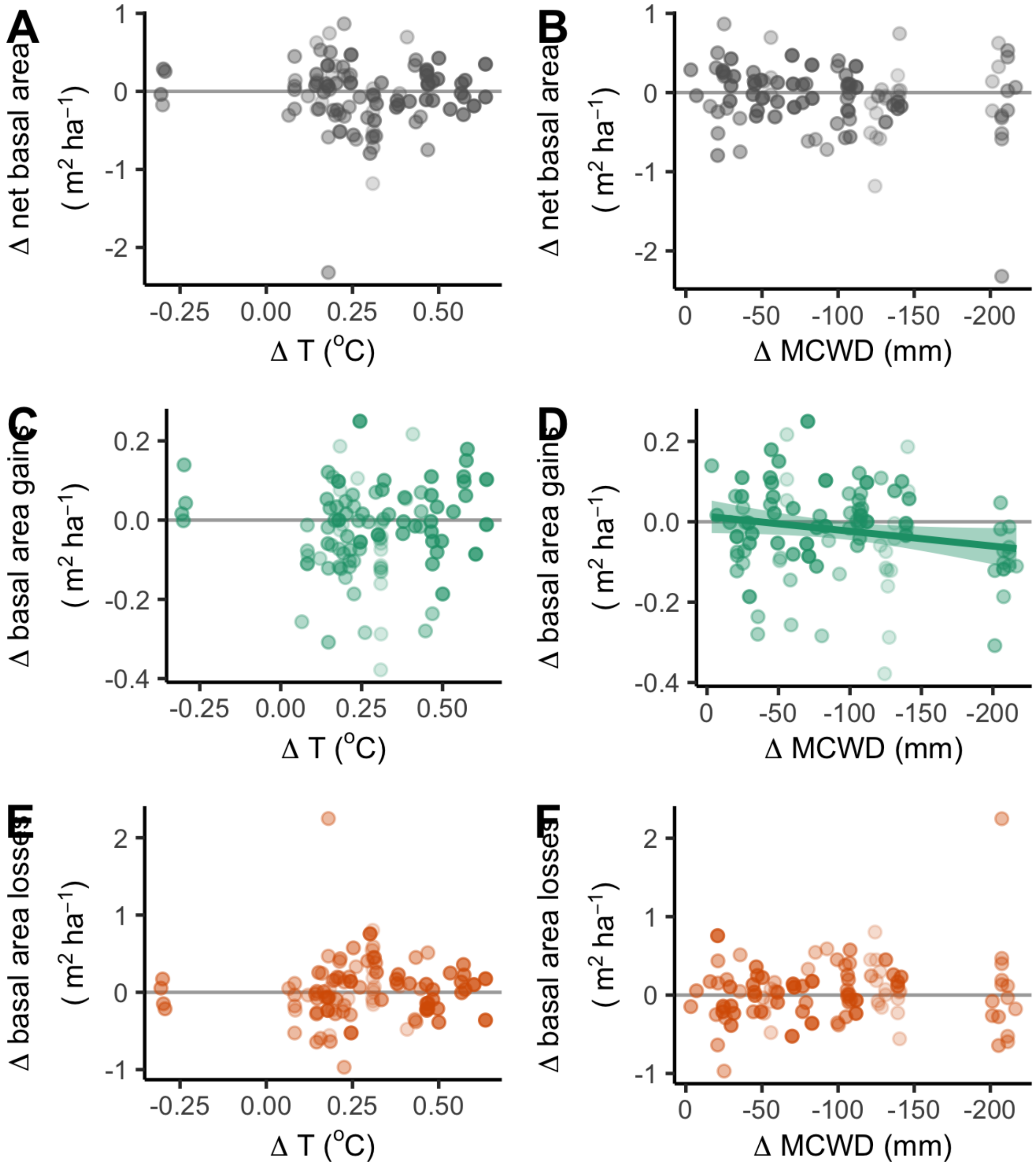

Fig. S12. Figure 2 reproduced using basal area, i.e., with no allometric assumptions.

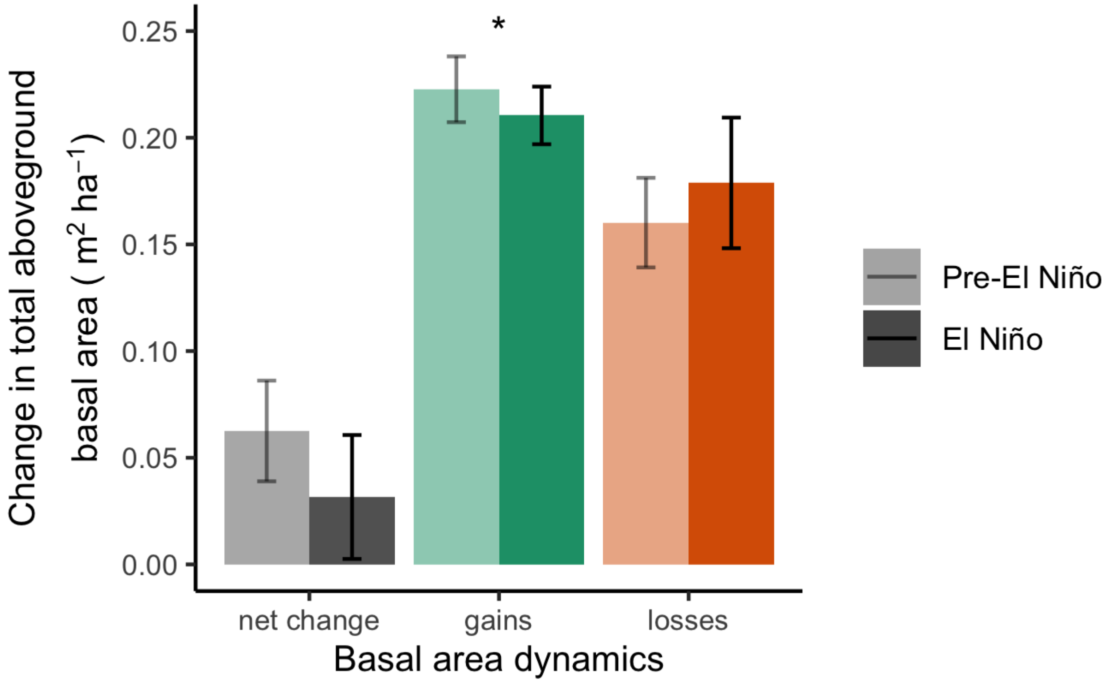

Fig. S13. Figure 3 reproduced using basal area, i.e., with no allometric assumptions. Gains significantly decrease by 5.5 % (paired t-test, * indicates p≤0.05).

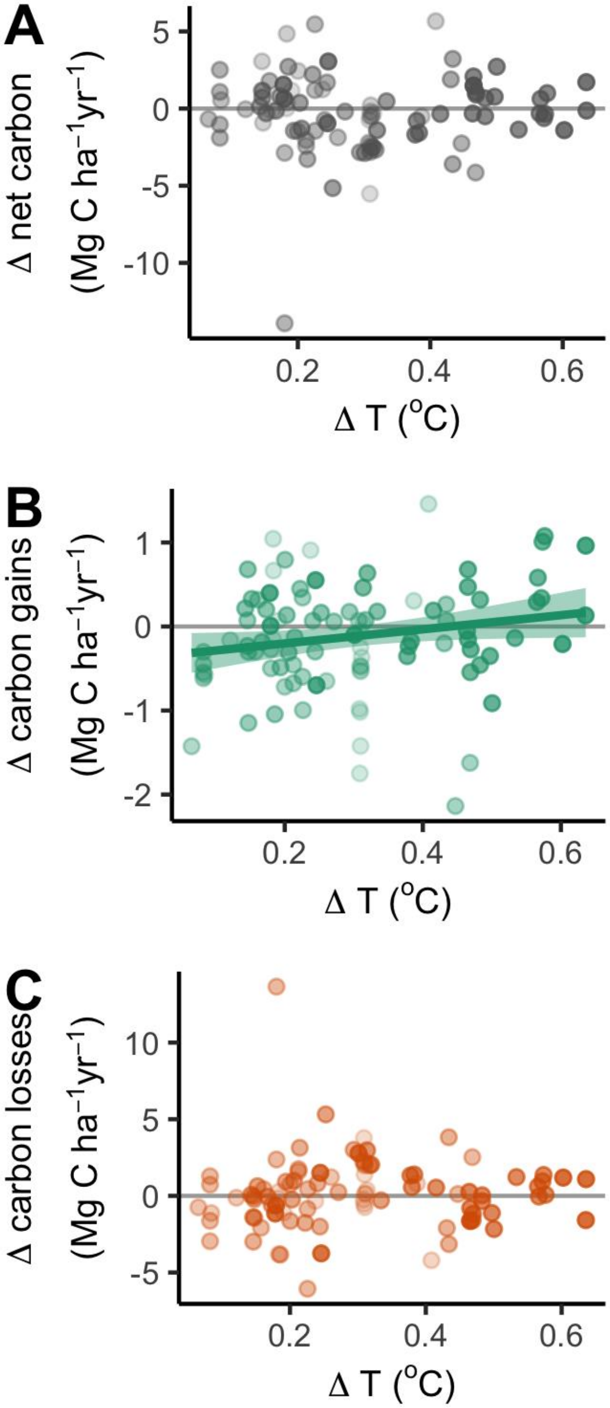

Fig. S14. Figure 2 reproduced excluding the four plots that did not show an increase in temperature.

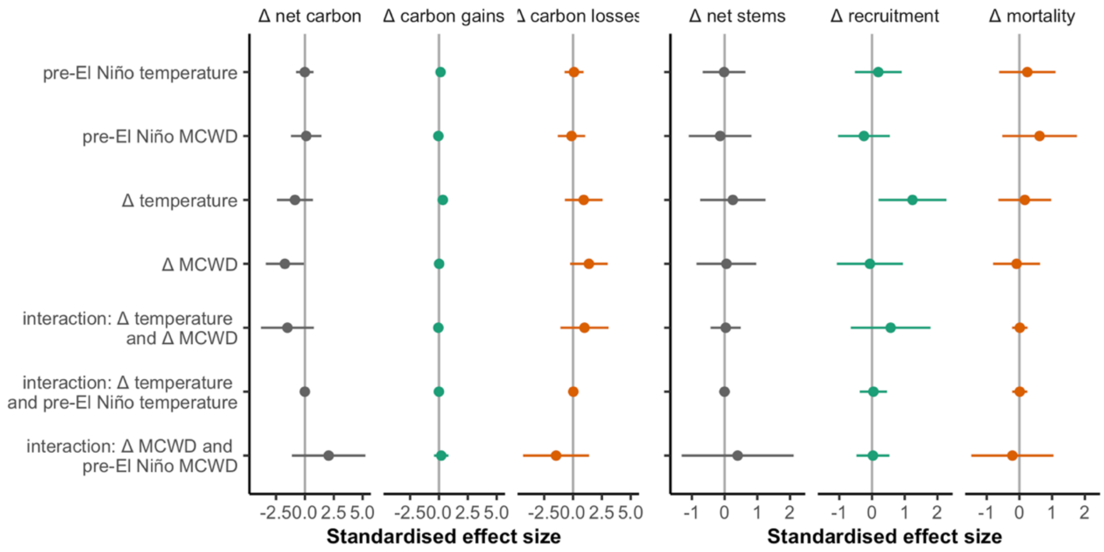

Fig. S15. Figure 4 reproduced excluding the four plots that did not show an increase in temperature.

Table S7. Summary of climate anomaly studies in tropical forests.
Values in bold indicate significant changes, as reported.

	**Climate anomaly type**	**Region**	**Lat. range** (º)	**Long. range** (º)	**Sampling approach**	**n plots**	**Mean plot size** (ha)	**Mean anomaly interval** (yrs)	**Change in net aboveground live biomass carbon** absolute | relative ($Mg\ C\ ha^{-1}\ yr^{-1}$ | %)	**Change in carbon gains change** absolute | relative ($Mg\ C\ ha^{-1}\ yr^{-1}$ | %)	**Change in carbon losses** absolute | relative ($Mg\ C\ ha^{-1}\ yr^{-1}$ | %)
Phillips et al. 2009	2005 Atlantic sea-surface temperature associated drought	Amazonia	-17 to 6	-78 to -52	Emergency remeasurement	55	1.7	2.0	**-0.73 | -0.5**	Figure not reported; No significant change reported.	Figure not reported; **Significant increase reported.**
Feldpausch et al. 2016	Atlantic sea-surface temperature associated drought and moderate 2010 ENSO event	Amazonia	-19 to 11	-78 to -52	Emergency remeasurement	97	1.2	2.3	**-0.81 | -0.6**	**-0.23 | -0.2**	**+0.66 | +0.5**
Qie et al. 2017	Very strong 1997-98 ENSO event	Borneo	-1 to 6	110 to 118	Post-hoc analysis	19	1.3	3.5	-1.44 | -0.7<br>**-2.03** | **-1.0** *	+0.10 | +0.1	**+1.62 | +0.8**
This study	Very strong 2015-16 ENSO event	Africa	-2 to 7	-8 to 25	Emergency remeasurement	100	0.9	2.7	-0.29 | -0.2	-0.11 | -0.1	+0.18 | +0.1

*Decline in sink during ENSO compared to before (above) and after (below) the ENSO event.

SI References

1. A. R. Martin, M. Doraisami, S. C. Thomas, Global patterns in wood carbon concentration across the world's trees and forests. *Nat. Geosci.* **11**, 915–920 (2018).
2. J. Talbot, *et al.*, Methods to estimate aboveground wood productivity from long-term forest inventory plots. *For. Ecol. Manage.* **320**, 30–38 (2014).
3. T. S. Kohyama, T. I. Kohyama, D. Sheil, Estimating net biomass production and loss from repeated measurements of trees in forests and woodlands: Formulae, biases and recommendations. *For. Ecol. Manage.* **433**, 729–740 (2019).
4. O. L. Phillips, *et al.*, Drought Sensitivity of the Amazon Rainforest. *Science (80-.).* **323**, 1344–1347 (2009).
5. T. R. Feldpausch, *et al.*, Amazon forest response to repeated droughts. *Global Biogeochem. Cycles*, 1–19 (2016).
6. L. Qie, *et al.*, Long-term carbon sink in Borneo's forests halted by drought and vulnerable to edge effects. *Nat. Commun.* **8**, 1966 (2017).
